# Profiling Transcriptomic Changes Across Frontal Cortical Subregions in Major Depressive Disorder

**DOI:** 10.21203/rs.3.rs-7349413/v1

**Published:** 2026-10-08

**Authors:** Artemis Iatrou, Adrien Gigliotta, Vijetha Balakundi, Rahul Bharadwaj, Chris Chatzinakos, Natalie Matosin, Corina Nagy, Clara Snijders, Gustavo Turecki, Boyan Leng, PsychENCODE PTSD Brainomics Project, Daniel Weinberger, Thomas Hyde, Charles Nemeroff, Kerry Ressler, Joel Kleinman, Nikolaos Daskalakis

**Affiliations:** Boston University; Boston University; Boston University; Lieber Institute for Brain Development; SUNY Downstate Health Sciences University; The University of Sydney; McGill University; McLean Hospital; McGill University; McLean Hospital; Multi-institute; Lieber Institute for Brain Development; The Lieber Institute for Brain Development; University of Texas at Austin Dell Medical School; McLean Hospital; The Lieber Institute for Brain Development; McLean Hospital, Harvard Medical School

## Abstract

Major depressive disorder (MDD) involves frontal cortical dysfunction, with emerging evidence implicating gliovascular and immune mechanisms, yet its transcriptional signatures across subregions, cell-types, and molecular layers remain poorly defined. Here, we integrated bulk-tissue RNA-seq data from dlPFC, mPFC, and dACC (n = 286) to identify a conserved core transcriptional profile, anchored in downregulated interneuron markers, and upregulated immune and vascular genes, spatially enriched in leptomeninges and white matter. Concordantly, multiomic factor analysis revealed immune activation, stress-response, and accelerated aging in MDD. Analysis of snRNA-seq data from dlPFC, mPFC, and oFC (863,511 nuclei; n = 204) revealed most pronounced MDD signatures in excitatory neurons, accompanied by coordinated disruption of glucocorticoid receptor machinery across cell-types, including *FKBP5* upregulation. Glial, immune, and perivascular populations exhibited signatures of oxidative stress, ECM remodeling, and reduced neuronal support, and greater concordance with bulk-tissue MDD signals than neurons. A global reduction in cell-to-cell communications in MDD revealed a shift from homeostatic neuronal signaling toward endothelial and myelination-associated pathways. Ephrin-Eph signaling was consistently dysregulated, implicating disrupted axonal guidance, synaptic remodeling, and neurovascular integrity. Notably, MDD signatures were shared between MDD-only and PTSD comorbid cases at bulk-tissue analyses, while diverging at single-cell resolution. Together, these findings reframe MDD as a distributed frontal cortex disorder, driven by disrupted neuroglial and neurovascular communication.

## Introduction

Major depressive disorder (MDD) is a leading cause of disability worldwide, severely impacting social, occupational, and cognitive functioning. Beyond its established effects on well-being, mood, reward processing, appetite and sleep, MDD is increasingly recognized as a systemic disorder^1^, with peripheral pathologies, such as vascular dysfunction, chronic inflammation^2^, and insulin resistance. Its heterogenous symptomatology leads to frequent co-diagnosis with other stress-related disorders like post-traumatic stress disorder (PTSD)^3^, and it often co-occurs in the context of lifetime adversity, including childhood trauma and substance abuse^4,5^. Within the central nervous system, MDD affects key regions, including the prefrontal cortex (PFC) and limbic system, with neuroimaging studies highlighting structural and functional changes in the PFC, potentially linked to disruptions in glutamatergic and GABAergic neurotransmission^6–8^.

Genomic and postmortem brain transcriptomic studies have associated MDD with dysregulation of the adaptive response to chronic stress and hint towards a complex interplay of genetic and environmental factors contributing to disease pathophysiology^9^. While the role of GABAergic interneurons, particularly SST-expressing subtypes^10–12^, has been extensively studied, emerging single-cell (sc) transcriptomic research has revealed critical contributions from non-neuronal cell-types including astrocytes and oligodendrocytes, and most recently endothelial cells^12,13^. Dysregulation of immune-related pathways, axonal guidance mechanisms, and neurovascular integrity have gained attention as novel components of MDD pathophysiology^14–16^. Moreover, a fragilized blood brain barrier (BBB) has been suggested as parallel process to the aggravated disease symptomatology^17^, suggesting a key role for vascular and immune cells. Despite these advances, the interplay between neuroglia, neuro-immune, and neurovascular systems remain poorly understood.

A major limitation in the field is the lack of integration of transcriptomic signatures across cortical subregions^18^. Although prior postmortem studies—including our own, which demonstrated heightened susceptibility of the medial PFC to stress-related transcriptional changes relative to other brain regions—have provided important region-specific insights, they have largely treated these regions in isolation^12,18,19^. This approach may obscure shared molecular signatures that reflect common vulnerability to stress across cortical circuits. A unified, cross-regional framework is therefore needed to identify consistent molecular alterations that are not limited in anatomical boundaries while retaining cell-type specificity.

Here, we address this gap by integrating new and reanalyzed cohorts of individuals with MDD and neurotypical controls, combining bulk-tissue and single-nucleus RNA sequencing to construct a unified transcriptional landscape of the frontal cortex (FC). By leveraging these cross-modal/regional analyses, we aim to (1) explore stress-related transcriptomic vulnerabilities across cortical subregions, (2) delineate cell-type specific contributions of MDD pathophysiology through single-nucleus transcriptomic analysis of cortical regions and (3) ultimately shift the focus from predominantly neuron-centric studies to investigation of molecular and functional contributions of non-neuronal cell-types, with the goal to enhance the mechanistic underpinning of the MDD pathology. To our knowledge this is the first study to analyze a FC-aggregated mRNA expression across both bulk-tissue and single-cell resolution in MDD, providing a novel framework to investigate neuroglial, neuro-immune and neurovascular connections in the disorder.

## Results

### Transcriptomic profiling of the FC in individuals with MDD

RNA sequencing (RNA-seq) was used to characterize the transcriptomes of three FC subregions from postmortem tissue of individuals diagnosed with MDD and from neurotypical controls (NC; **Sup Table 1A-B**): the ventromedial PFC [(v)mPFC, Broadmann area 12), the dorsal anterior cingulate cortex (dACC, BA 24) and the dorsolateral PFC (dlPFC, BA 9/46) (Fig. 1A). MDD diagnosis was defined based on a primary Axis I diagnosis of MDD (MDD only) or a secondary diagnosis of MDD in individuals with a primary diagnosis of PTSD (PTSD-comorbid), resulting in an overall MDD-control ratio of approximately 2:1 for MDD. Across subregions, we examined a variable number of individuals (N_mPFC_ = 283, N_dACC_ = 143, N_dlPFC_ = 142) with 138 individuals overlapping across all subregions (N_unique individuals_ = 286). Cohorts were balanced for sex and approximately age-matched between groups and were predominantly of European ancestry (~ 85%; **Sup Table 1C**).

We applied two complementary analytical approaches to characterize MDD-associated differential gene expression (DGE) across the FC and capture both shared (cross-region) and region-specific MDD transcriptional signatures. First, we implemented an aggregated model across cortical subregions (FC-aggregated), incorporating sample as a random effect and adjusting for sex, age at death, ancestry, brain region, RNA integrity number (RIN), postmortem interval (PMI), mitochondrial reads, and inferred cell-type proportions (Fig. 1B). This model showed that the highest variance is explained by the donor effect (average 23.8% of variance explained), while the region only explained an average of 6.6% **(Fig. S1A)**. Second, we performed region-specific linear models for each subregion, adjusting for the same covariates **(Fig. S1B-D).**

MDD signals from the aggregated model included a total of 2,936 unique differentially expressed genes (DEGs) comprising 1,584 upregulated and 1,352 downregulated genes passed a false discovery rate (FDR) threshold of 5% **(Sup Table 2A)**. The most significantly upregulated genes were *EMP3* (FDR-adjusted P-value = 1.8e-07), *CLU* (P_adj_ = 4e-07), *LRP10* (P_adj_ = 1e-06), *EPHA2* (P_adj_ = 1e-06), while the largest effect sizes were observed for *IL1RL1* (log_2_FC = 1.46, P_adj_ =0.002) and *SERPINA3* (log_2_FC = 1.4, P_adj_ =0.002). In contrast, the most significantly downregulated gene was *DLX6-AS1* (P_adj_ = 1.2e-07), with the largest magnitude decrease observed for *LINC00996* (log_2_FC = −0.93, P_adj_ = 1.6e-03). Notably, several of the top downregulated genes are established interneuron markers, including *CORT* (P_adj_ = 2e-04), *SST* (P_adj_ = 1.4e-03), and *CRH* (P_adj_ = 2.2e-05).

The subregion-specific analysis identified 761 DEGs (up 505, down 256) in the mPFC, 547 DEGs (up 347, down 200) in the dlPFC, and 816 DEGs (up 307, down 509) in the dACC **(Sup Table 2B-D)**. Across the FC-aggregated analysis and the three region-specific analyses, which shared approximately 22,000 expressed genes, the overlap between DEGs was substantial: 83% of mPFC DEGs (633 genes), 82% of dlPFC DEGs (450 genes), and 70% of dACC DEGs (579 genes) were also detected in the FC analysis, with effect size concordance of over 73% across the regions (Fig. 1C). In this study, correlation coefficients were interpreted as follows: 0–0.1, negligible; 0.1–0.3, weak; 0.3–0.6, moderate; and > 0.6, strong. Although genome-wide ranked correlations of differential expression effect sizes between individual subregions (e.g., mPFC, dACC, dlPFC) was moderately high (ρ = 0.5–0.65), for the subset of FDR genes the correlations were strong (ρ = 0.98–0.99) **(Fig. S1E)**. This indicates that the most robust MDD-related transcriptional changes are largely conserved across FC subregions, with evidence of region-specific modulation. Furthermore, comparing the genome-wide signatures from the subregion-specific analyses to the FC-aggregated model revealed strong overall correlations (ρ > 0.68). For genes that were significantly (FDR) associated with MDD in both approaches, their rankings were nearly identical (ρ > 0.98) (Fig. 1D, **Fig. S1F**). These findings suggest that the aggregated model effectively captures the core transcriptomic features of MDD across FC subregions. We also leveraged publicly available results from Girgenti et al., 2021^18^ to compare MDD-specific signatures from two additional cortical subregions, the orbitofrontal cortex (oFC; BA 11) and subgenual PFC (sgPFC; BA 25) (**Fig. S2A**). We observed modest correlations at the nominal level (ρ ~ 0.3) between the FC-aggregated and region-specific analyses of oFC and sgPFC, which became high (ρ ~ 0.7) when focusing on overlapping FDR genes.

To evaluate the robustness across clinically relevant subgroups of MDD-associated transcriptional alterations, we performed two complementary sets of secondary analyses (Fig. 1E). First, we conducted stratified subgroup analyses to assess whether disease-associated signatures were preserved across clinically relevant subsets, including primary MDD without PTSD comorbidity (MDD-only), PTSD-comorbid with MDD (PTSD-comorbid), sex-stratified MDD cases, and low-substance/toxicology-negative MDD cases (Fig. 1F). Second, we performed sensitivity analyses to evaluate the potential influence of childhood trauma exposure, and substance use as covariate on the observed transcriptional patterns (**Fig. S2B).**

Across all stratified subgroup analyses, FC-aggregated transcriptional signatures remained highly concordant with the primary MDD model, supporting the robustness of the core disease-associated signal (Fig. 1F; **Sup Table 2E)**. The strongest concordance was observed for the MDD-only and PTSD-comorbid analyses (ρ = 0.91 and 0.90, respectively), while sex-stratified results were more divergent. Female-specific analyses showed reduced concordance based on the aggregated model (ρ ~ 0.64 in FC), decreasing to moderate (ρ = 0.47) in dACC and mPFC, with the dACC exhibiting the most distinct profile in the female-specific analysis and mPFC in the male-specific one. Direct comparisons between male and female MDD cases further highlighted sex-specific effects, with low cross-sex correlations (ρ = 0.08 in the FC-aggregated model and ρ = 0.28 in the dlPFC), suggesting sex-dependent transcriptional variation. Notably, only a small proportion of FDR-significant DEGs mapped to sex chromosomes (2.9% in females and 3.2% in males). Direct comparison between the MDD-only and PTSD-comorbid analyses revealed strong concordance across FC-aggregated and region-specific signatures (ρ = 0.60–0.81), indicating that bulk-level transcriptional alterations largely reflect shared disease-associated signatures between primary MDD and PTSD-comorbid cases. To evaluate whether the contribution of substance use history severity (polysubstance and opioid) further affected the transcriptomic profile we performed an analysis on the MDD cases with no history of substance use/abuse/dependence vs controls and we reported moderately strong genome-wide correlation of effect sizes of 0.64, suggesting some influence, although interpretation is limited by the substantially smaller subgroup sample sizes (96 NC vs 26 MDD).

Sensitivity analyses accounting for childhood trauma exposure, and polysubstance/opioid use produced highly similar transcriptional profiles relative to the primary model, with genome-wide correlations of effect size with the FC model averaging around 0.8 (CT: 0.85, Substance: 0.78; **Fig. S2C)**, further supporting the stability of the observed MDD-associated signatures across multiple clinical covariates. DGE results for all secondary and sensitivity analyses can be found in **Supplementary Table 3**.

Together, these results indicate that MDD is associated with a conserved core transcriptional program across FC subregions, encompassing interneuron, synaptic, and axonal guidance gene signatures, with the most robust disease-associated genes showing near-identical effect-size rankings regardless of the subregion or analytical framework employed. This core signal was robust to clinical heterogeneity with bulk-level transcriptional profiles largely shared between MDD-only, PTSD-comorbid MDD, and models accounting for early-life adversity and substance (ab)use, potentially reflecting a shared transcriptional signature of stress-related psychopathology. In contrast, biological sex moderated these transcriptional patterns, with specific FC subregions exhibiting the greatest sex-dependent divergence.

### Functional, Spatial and Positional Enrichment of MDD signatures

To link the MDD transcriptomic profile to functional pathways, we performed gene set enrichment analysis (GSEA) based on gene ontology biological processes (GO:BP) (**Sup Table 4A-D**). Among all the significantly positively enriched pathways in the FC-aggregated analysis, eight major biological themes emerged based on semantic similarity of terms, including negative regulation of vascular cell proliferation, positive regulation of TOR signaling, developmental maturation, extracellular matrix (ECM) disassembly, metabolism, regulation of immune response, regulation of insulin secretion and vesicle localization (Fig. 2A). These pathways showed strong concordance across regions, with all top positively enriched pathways reaching statistical significance and exhibiting consistent directionality in each subregion. The negatively enriched pathways were primarily associated with cell migration, DNA recombination, Fc epsilon receptor signaling, intraciliary transport, and disrupted cell processes (Fig. 2A). While these pathways demonstrated consistent negative enrichment scores across regions, their statistical significance was less uniform. This asymmetry was further supported by correlation at pathway-level analyses, which showed stronger cross-region concordance for positively enriched pathways compared to negatively enriched pathways **(Fig. S3A).** Representative top upregulated and downregulated pathways can be found in **Fig. S3B** exhibiting patterns of immune response signaling disruption, axon guidance and synaptic dysregulation, and endothelial cell apoptosis, and synaptic processes which suggested the key involvement of non-neuronal-driven mechanisms in MDD. To examine whether the observed sex-stratified transcriptional differences were also reflected at the pathway level, we also performed pathway enrichment analyses separately in male and female MDD cases, and female-specific DEGs showed stronger enrichment for immune-related processes, while male-specific DEGs demonstrated greater enrichment for pathways related to proteostasis, including apoptotic signaling, autophagy, and protein folding (**Sup Table 4E**). Together, these findings suggest partially distinct molecular response programs between sexes in MDD, with the more distinct overall female-specific profile likely reflecting both reduced power and genuine sex specific transcriptional variation^20^.

Given that many of the non-neuronal cell-types-particularly glial and vascular populations-are enriched in specific cortical layers such as the upper layers (L) or white matter (WM), we next sought to spatially register MDD-associated transcriptomic changes across the dlPFC column (leptomeninges, L1–6, WM)^21^. We observed consistent enrichment of these signatures in the leptomeningeal space and Layer 1 in the FC-aggregated analysis and all region-specific analyses (Fig. 2B; **Sup Table 5A)**. Additionally, the FC-aggregated disease signal showed enrichment in deeper WM compartments, a pattern that was directionally consistent across regions but reached statistical significance only in the dACC (Fig. 2B). To validate the spatial registration results using an orthogonal approach, we tested for enrichment of region-specific bulk-tissue MDD DEGs among cell-type marker genes derived from our integrated single-nucleus atlas introduced below **(Fig. S3C).** Across all cortical regions, MDD-associated transcriptional changes were consistently enriched for non-neuronal cell-type markers and depleted for neuronal markers, mirroring the Layer 1 and WM enrichments observed in the spatial analyses. These findings support the robustness of the spatial registration results and suggest that the weaker dACC spatial signal likely reflects technical limitations of the registration framework rather than an absence of the underlying biological signal.

Exploration of the chromosomal colocalization of the MDD transcriptomic profile showed that the FC-aggregated model increased power to detect enrichment across multiple genomic loci **(Sup Table 5B)**. Within the FC-aggregated model, the strongest enrichment signals were observed at Chr19p13 (NES = 2.83, p.adj = 1.2e-39) and Chr9q34 (NES = 2.85, p.adj = 8.9e-23), followed by Chr17q25 (NES = 2.68, p.adj = 3.5e-18) and Chr1p36 (NES = 2.37, p.adj = 7.4e-17). Additional significantly enriched loci included Chr11q13, Chr17q21, Chr6p21, Chr22q13, Chr16p13, and Chr20q13 (Fig. 2C). Notably, several of these loci showed consistent enrichment across all analyses, including Chr16p13, Chr17q21, Chr17q25, Chr1p36, Chr1q21, Chr20q13, and Chr6p21, indicating robust cross-regional convergence of transcriptional signal.

Seeing the differential enrichment of genomic loci in our DGE results, we aimed to link the transcriptomic-based cortical signature with genes associated with psychiatric disease risk. We therefore performed MAGMA gene-set enrichment analyses using GWAS summary statistics for MDD^22^, PTSD^23^, schizophrenia (SCZ)^24^, and bipolar disorder (BIP)^25^. Across cortical regions, disorders, and DEG directions, no associations survived correction for multiple testing, with effect size estimates generally small and inconsistent across traits and subregions (**Fig. S3D; Sup Table 5C).** These findings are consistent with the emerging understanding that genetic risk variants and disease-state transcriptional changes often reflect distinct biological processes^26–28^.

We conducted a summary data-based Mendelian randomization (SMR) using expression quantitative trait loci (eQTL) from BrainMeta (N = 2,865)^29^ and the latest PGC-led MDD GWAS^22^ (N_cases_= 412,305) to assess whether transcripts and traits are associated due to shared causal variants. Among the SMR-emerged 328 eGenes (Padj < 0.05, HEIDI p > 0.05; **Fig. S3E**, **Sup Table 5D**), i.e. genes with significant colocalization between a cis-eQTL signal and a GWAS signal, we reported an overlap of 40 eGenes with our DEGs, with 24 showing concordant regulation (Fig. 2D). This overlap did not exceed what would be expected by chance (permutation P = 0.902; observed = 40, expected = 47.5), indicating that the gene-level overlap between SMR and DGE is not globally enriched beyond chance, although a subset of genes shows biologically convergent dysregulation across both layers of evidence, namely *NCOA1, PBRM1* and immune-related genes like *C1QTNF7*, *RELT*, *SPATA2*, *TRIM8* (Fig. 2D).

We further applied transcriptome-wide association models (TWAS) trained in 2,365 cortical samples^30^ to the most recent depression GWAS, using JEPEGMIX3-P^31^. SMR and TWAS gene sets showed highly significant overlap (186 shared genes; permutation P < 1×10^−4^), indicating substantial convergence between genetically regulated expression signals identified by the two complementary approaches. Intersecting these results with our DGE analysis identified 25 genes supported by all three methods (*ARAP3, CHSY1, CTTNBP2, FAM178B, GOLGA1, GRAMD1A, INAFM1, NCKIPSD, NCOA1, NSFL1C, PBRM1, POLR1E, PRDM11, PSTK, RELT, RNF4, RPLP0, SORBS3, SPATA2, TAL1, TKT, TRIM8, USP24, ZNF462, ZNHIT1*), associated with biological processes implicated in MDD pathophysiology (Fig. 2D). These include but are not limited to stress-axis and glucocorticoid receptor (GR) signaling (*NCOA1, PBRM1*), synaptic scaffolding and dendritic spine maintenance (*CTTNBP2*^32^, *NCKIPSD*), mitochondrial function and oxidative stress defense (*TKT*), ECM and perineuronal net regulation (*CHSY1, SORBS3*), and immune and ubiquitin-proteasome signaling (*TRIM8, SPATA2, RELT, RNF4, USP24*). CTTNBP2, the most extensively characterized among these, is a postsynaptic density protein linked to dendritic spine stability and synaptic plasticity, functions known to be impaired in depression^33^, and its association with MDD was recently highlighted in other subcortical brain regions. NCOA1, a coactivator of the GR that directly facilitates GR-dependent transcriptional activation^34^, provides a link to the stress-response signature, while PBRM1 is a core subunit of the SWI/SNF chromatin remodeling complex which has been implicated in stress-induced behavioral adaptation in mice^35^.

Altogether, the transcriptomic landscape of MDD in the FC implicated pathways related to inflammation, ECM remodeling, axonal guidance, apoptosis, and dysregulation of synaptic plasticity. The captured signals were spatially concentrated to non-neuronal rich cortical domains. Critically, a subset of these transcriptional changes is genetically grounded: integration with SMR and TWAS analyses identified 25 genes at the intersection of disease-state expression and genetically regulated programs, spanning synaptic scaffolding, mitochondrial resilience, and protein homeostasis. These findings suggest that MDD-associated FC transcriptome is not a mere collection of region-specific noise, but rather a coherent, spatially organized and partially genetically regulated signature.

### Multiregional and multi-omic views

We employed Multi-Omics Factor Analysis (MOFA) to reduce dimensionality by inferring 30 latent factors that capture certain variance (R^2^) of the transcriptomic layers in each brain region and proteomic and methylomic layer in the mPFC across the 138 common samples (Fig. 2E). We observed factors focusing on a single omic feature, i.e. Factor 1 ~ methylation, Factor 5 ~ protein expression, Factors 2, 3, 6 ~ the RNA-based input from each brain region. Variance across omics for the mPFC was captured by Factor 17, a“multi-omic” factor and variance within RNA across regions was captured by Factors 4 and 15, setting them as the “cortical” factors (Fig. 2E). Finally, Factor 13 explained variance across all regions and omics, suggesting its function as a “global” factor. Further exploration into Factor 13 showed that it is highly correlated with chronological (ρ = −0.89) and Horvath methylation-based age (ρ = −0.84) (Fig. 2F, **Fig. S2F)**, indicating an inverse relation between the factor score and aging. The methylation age of the controls was strongly anti-correlated with Factor 13 (ρ = −0.91). In the MDD subgroup, the correlation remained strong (ρ = −0.84) (Fig. 2F), but showed a steeper slope (standardized major axis, p = 0.015), suggesting accelerated aging patterns and its potential application as a cortical biological clock able to distinguish the diagnostic nuances. Notably this factor was functionally enriched in cell-cell signaling, inflammatory response, neuron development, and vascular development pathways across the regions and was weakly positively associated with glial cell-type proportions and negatively with immune cell-type proportions (|rho| = 0.15) (**Fig. S3G; Sup. Table 5F**). Factor 4 scores were significantly higher in the MDD group versus the control (t = −5.8, p = 5.2e-08, Fig. 2G). Indeed, this remained the case with pairwise comparisons showing Factor 4 scores were higher in MDD-only relative to controls (p.adj = 2.2e-04) and in PTSD-comorbid relative to controls (p.adj = 4.5e-04), while MDD-only and PTSD-comorbid did not differ significantly (p.adj = 0.24) (**Fig. S3H).** This suggests that Factor 4 captures shared transcriptional alterations associated with stress-related psychopathology. Functionally, Factor 4 was enriched in immune response, response to stress, cell death, and response to corticosteroids pathways, and had weak associations with immune cell-type deconvolved proportions (rho = 0.15) (**Fig. S3G; Sup. Table 5E).** Factor 17 showed a low correlation with the ancestry PCs (ρ ~ 0.19) (**Fig. S3F**).

These results indicate that the MDD cross-cortical pathophysiology can be captured by a distinct factor that is related to immune activation, stress response and cell death. Further, a second factor representing integrative modeling across cortical subregions and omics is tightly linked to methylation age and shows that MDD is characterized by accelerated aging.

### Profiling the MDD cortex in single-cell resolution

Based on the bulk-tissue RNA sequencing results, which indicated robust MDD-associated transcriptional changes with limited regional specificity, we next focused on exploring common features between three cortical subregions, dlPFC, mPFC and orbitofrontal FC (oFC), in single-cell resolution.

We used two in-house generated (one new and one published^36^) and two publicly available datasets^37–39^ which we reanalyzed and integrated to compose the working dataset for this study (study1_McLean, N = 46, study2_McGill, N = 70, study3_MPI = 39, study4_McLean_mPFC = 49). Assessing the demographic variability of each dataset, we observed a significant association of diagnosis with age (T = 2.5, p = 0.01), justified by the fact that the study2_McGill MDD samples were all suicide completers contributing to a younger age of death at the MDD group (**Sup Table 6A-B**). The combined dataset included 106 MDD vs. 98 NC, making it the largest single cell MDD analysis so far. There was a minimal overlap of 3 samples across the single cell brain regions (dlPFC-mPFC), and a total overlap of 60 samples with the bulk-tissue working dataset.

To minimize the influence of region-specific subpopulations, we first clustered cell-types (e.g., astrocytes) within each dataset, removed clusters not corresponding to the target cell-type, and then performed integration, harmonization, and downstream analyses on the retained common cell populations across regions. This approach resulted in a dataset containing 863,511 single nuclei (Fig. 3A), that clustered in 7 main clusters in order of abundance: excitatory neurons (Ex; 47%), inhibitory neurons (Inh; 21%), oligodendrocytes (oligo; 14%), astrocytes (astro; 10%), immune cells (4.3%), oligodendrocyte precursor cells (OPC; 4%) and cells of the perivascular space (0.6%) (all cell classes markers are provided in **Sup Table 7A)**. We assessed cell-type proportion differences between MDD and NC while adjusting for age, sex, ancestry, study, chemistry version, tissue, and donor, and we did not detect any significant cell-type proportion (Fig. 3B **showing unadjusted proportions**). Variance partition analysis indicated that the majority of variance was explained by donor and the study effects (**Fig. S4A)**. When considering primary diagnoses, compared to NC, MDD-only samples showed a modest reduction in macrophages (T = −2.91, Bonferroni-adjusted p = 0.045), while PTSD–comorbid samples showing no significant changes across cell-types **(Fig. S4B)**. Sex differences were modest and did not survive Bonferroni correction **(Fig. S4C)**. Finally, regional effects were most pronounced in astrocytes, which were significantly more abundant in the mPFC (T = 4.06, Bonferroni-adjusted p = 7.8e-4) and showed a nonsignificant trend in the oFC (T = 2.53, Bonferroni-adjusted p = 0.133) in comparison to dlPFC **(Fig. S4D)**. Overall, these results indicate that while some cell-types show subtle changes across disease status, most proportional differences are small once covariates are accounted for.

Further sub-clustering of the main clusters allowed us to distinguish different cell subclasses and more nuanced cell-types in the general Immune and Perivascular clusters (Fig. 3A, 3C). All cell subclasses markers are provided in **Sup Table 7B** and functional annotations of the classes in **Sup Table 7C**). Within the Excitatory neurons, we had nuclei belonging to 10 subclasses, each characteristic of specific cortical layer and projection. Similarly, we observed 5 inhibitory subclasses, annotated by the main interneuron markers, namely, LAMP5, SST, PVALB, VIP, and RELN. Oligodendrocytes formed 3 distinct clusters, characterized by their top markers (Fig. 3A) while OPC nuclei did not form highly distinct clusters. Further subclustering of the astrocyte cluster revealed four main populations previously seen in Serrano-Pozo et al.^40^; a homeostatic subclass (astH0; 48,054 cells), a reactive cluster (astR; 14,286 cells), an inflammatory cluster (astInf; 8,567) and an astrocyte cluster expressing some neuronal markers (astNeu; 14,477 cells) (Fig. 3A). In the immune cell cluster, we detected microglia (homeostatic and disease-associated, Micro, 35,215 cells), macrophages (1,052 cells), and T-cells (500 cells) (Fig. 3A). Finally, in the perivascular microenvironment, we identified endothelial cells more closely resembling capillaries (Endo, 1,248 cells), mural cells [pericytes and smooth muscle cells; Mural, 2,606 cells] and fibroblasts (mainly perivascular and some rare meningeal subtypes, Fibro, 1,287 cells) (Fig. 3A) Expression levels of canonical markers for the most distinct cell-types can be seen in Fig. 3C.

We next evaluated the enrichment of MDD genetic risk in cell-type-specific gene sets using two complementary approaches: MAGMA gene-set analysis and stratified LD score regression (S-LDSC), applied to the latest depression GWAS^22^. The two methods showed strong convergence for three cell types; excitatory neurons (Ex; MAGMA p_bon_ = 2.6×10^−4^, S-LDSC p_bon_ = 6.8×10^−9^), inhibitory neurons (Inh; MAGMA p_bon_ = 3×10^−5^, S-LDSC p_bon_ = 4.6×10^−10^), and oligodendrocyte precursor cells (OPC; MAGMA p_bon_ = 1.6×10^−7^, S-LDSC p_bon_ = 7.5×10^−11^) all showed significant enrichment surviving Bonferroni correction in both analyses (Fig. 3D, **Sup Table 7E**). Astrocytes and Oligodendrocytes showed weaker enrichment reaching significance in S-LDSC (p_bon_ = 1.9×10^−7^) but not MAGMA (p = 0.071), Oligodendrocytes showed nominal enrichment in LDSC only (p = 2.3×10^−3^), while remaining cell types showed no significant enrichment in either analysis. These findings highlight a convergent genetic architecture implicating both neuronal populations and oligodendroglia precursors as primary cellular substrates of MDD genetic risk, with astrocytes as a potential secondary contributor. The lack of enrichment in endothelial and immune cell types suggests that the vascular and neuroinflammatory signatures observed at the transcriptional level may reflect downstream or environmentally driven processes rather than primary genetic liability. Applying the same analyses to PTSD GWAS^23^ data identified a highly similar pattern of enrichment, with excitatory neurons, inhibitory neurons, and OPCs surviving Bonferroni correction in both MAGMA and S-LDSC, though reflecting more of a neuronal genetic enrichment (**Sup Table 7F**).

Together, these analyses establish a high-resolution cellular atlas of the MDD FC comprising 863,511 nuclei across eleven major cell classes and their transcriptionally and functionally distinct subpopulations. Cell-type proportions were largely stable across diagnostic groups once covariates were accounted for, and partitioned heritability analysis identified inhibitory neurons and OPCs as the primary cellular substrates of MDD genetic risk.

### MDD-associated differential gene expression in the cortical cell-types

We then proceeded to investigate the MDD-specific differential gene expression (DGE) of the 11 cell-types. Our primary analysis focused on the generalized diagnosis contrast (gDx; MDD vs NC), which served as the main framework for downstream interpretation. In parallel, the study design enabled additional sub-analyses, including sex stratified MDD, and primary diagnosis-specific comparisons (MDD-only and PTSD-comorbid), as well as childhood trauma-targeted analysis. We performed cell-type specific DGE analysis, adjusting for biological (age, sex, ancestry) and technical (subregion, chemistry version, study batch) confounders. For cell-types comprising multiple transcriptionally distinct subclasses (excitatory and inhibitory neurons, astrocytes, oligodendrocytes, microglia, mural cells, and fibroblasts), we implemented a joint modeling approach in which subclass-specific expression profiles were analyzed simultaneously. In this approach, subclass-specific nuclei counts were incorporated by modeling subclass identity, allowing each subclass to contribute proportionally to the analysis, while accounting for repeated measurements from the same donor via a random intercept. Statistical inference was based on the main effect of MDD diagnosis, thereby capturing overall disease-associated transcriptional changes while controlling for subclass composition. For cell-types with more limited heterogeneity (macrophages, T cells, and endothelial cells), we performed DGE using a single-level model without subclass stratification. In these cases, we similarly accounted for repeated measures across donors using linear mixed-effects models when applicable, or linear models when tissue was contributed from unique samples. Differential expression results for each analysis and cell-type are provided in **Supplementary Tables 8A-B.**

The majority of FDR-significant signal was detected in excitatory neurons (Ex; 1,756 DEGs) and inhibitory neurons (Inh; 125) (Fig. 4A), followed by Astro (57), Oligo (41), Mural (15), OPC (9), Fibro (8), Micro (6), and Macro (3) (Fig. 4B). Among differentially expressed genes in Ex, *SEMA3A* (logFC = 0.48, p.adj = 2×10^−4^), a key regulator of axonal guidance and synaptic organization, was upregulated, while *PDGFD* (logFC = −0.63, p.adj = 6×10^−5^), a PDGF family member whose neuronal expression may influence paracrine signaling to neighboring vascular and glial cells, was downregulated. *FKBP5* (logFC = 0.38, p.adj = 0.02), a stress-responsive co-chaperone central to GR sensitivity and HPA axis regulation, was also upregulated, consistent with its established role in stress-related psychopathology and MDD^41^. Differential expression in inhibitory neurons highlighted genes involved in GABAergic development, synaptic organization, and neuronal excitability, including *DLX6-AS1* (logFC = −0.50, p.adj = 9.4×10^−11^), a lncRNA regulating DLX6-dependent GABAergic interneuron specification and implicated in psychiatric vulnerability; *EPHA4* (logFC = −0.28, p.adj = 5×10^−3^), a receptor tyrosine kinase governing synaptic and axonal remodeling; *TRPC3* (logFC = −0.37, p.adj = 5×10^−3^), a calcium-permeable channel contributing to neuronal excitability; and *KALRN* (logFC = 0.23, p.adj = 8×10^−3^), involved in dendritic spine morphology and synaptic plasticity. Together, these findings suggest altered inhibitory circuit structure and plasticity in MDD. Among glial populations, astrocytes showed dysregulation of neurotrophic and receptor signaling genes, including *SORCS1* (logFC = 0.70, p.adj = 0.01), a regulator of neurotrophin receptor trafficking and synaptic organization, and *SSTR2* (logFC = 0.69, p.adj = 0.01), a SST receptor whose upregulation may reflect compensatory changes in the context of well-documented somatostatin deficits in MDD. OPCs prominently featured *ITGB4* (logFC = 0.81, p.adj = 8×10^−3^), an integrin involved in ECM adhesion and myelination, and *EFNA5* (logFC = 0.77, p.adj = 8×10^−3^), an ephrin ligand also upregulated in oligodendrocytes, suggesting altered ECM adhesion and axon guidance signaling in OPC-axon interactions. Notably, the upregulation of EFNA5, a direct ligand for EphA4, in OPCs and oligodendrocytes paralleled the downregulation of its receptor EPHA4 in inhibitory neurons, pointing to convergent, potentially reciprocal disruption of ephrin-Eph signaling at neuron-glia interfaces. In immune populations, microglia exhibited altered expression of *CD83* (logFC = −1.1, p.adj = 7×10^−3^), a marker of microglial activation state, and *IRAK2* (logFC = −0.76, p.adj = 0.04), a kinase integral to innate immune and TLR signaling, consistent with altered neuroimmune activation in MDD. Among perivascular populations, fibroblasts demonstrated prominent dysregulation of ECM-associated and cytoskeletal genes, including *LAMA2* (logFC = −1.44, p.adj = 0.047), *CALD1* (logFC = −1.8, p.adj = 0.02), and *ARHGAP42* (logFC = −1.7, p.adj = 0.02), suggesting altered structural remodeling of the perivascular niche. Mural cells additionally showed changes in adhesion- and survival-related transcripts, including *ADGRB2* (logFC = 0.58, p.adj = 0.03), an adhesion GPCR involved in cell–cell communication and angiogenesis regulation, and *BCL2L2* (logFC = 0.53, p.adj = 0.04), an anti-apoptotic BCL-2 family member, pointing to potential shifts in mural cell survival signaling.

Beyond cell-type-specific changes, we identified genes with consistent differential dysregulation across multiple populations, including synaptic and calcium signaling mediators (*NRG1, SV2A, SV2B, CACNA1B, CACNA1D*), myelination-associated factors (*CLDN11*, *OPALIN*), and stress- and activity-responsive regulators (*GADD45A, FKBP5*), suggesting coordinated transcriptomic perturbations across the cortical circuit (**Fig. S4E).**

We next compared our findings to a recently published study by Hwang et al.^12^, which also provided cell-type-specific DGE results for the MDD analysis on top of the PTSD one which was the primary focus. In the Hwang paper, they used a comparable snRNA-seq framework but different approach to the DGE analysis where they employed Seurat-based MAST methods instead of a dream-based pseudobulk-tissue method in our case. Despite differences in cohort composition and methodology, we observed directional concordance, with 14 of 15 shared DEGs exhibiting matching directions of effect across datasets. Overlapping signal was concentrated in four cell-types: perivascular cells (n = 5 shared), inhibitory neurons (n = 4), astrocytes (n = 3), and excitatory neurons (n = 3), while no overlap was detected in OPCs, oligodendrocytes, or immune cells. Formal enrichment testing (Fisher’s exact test) indicated that the overlap was significant only in endothelial cells (fold enrichment = 2.21, permutation P = 0.018), while other cell types showed non-significant trends. Endothelial cells showed the highest proportional overlap from our dataset, with 5 of 23 significant DEGs (21.7%) replicated in the reference, including *LAMA2, ARHGAP42, PLEKHA5, RBMS3*, and *ZIC1*, all downregulated in both studies. In astrocytes, shared downregulation of *CSPG5* and *NCAN*^42,43^, both chondroitin sulfate proteoglycans integral to ECM composition and synaptic plasticity, was observed, reinforcing emerging evidence for glial ECM disruption in psychiatric disorders.

The observation that among the DEGs were *FKBP5* and genes transcribing HSP70 led us to examine the overall expression profile of the GR co-factors in each cell-type (**Fig. S4F).** We saw that *FKBP5* was overall upregulated (nominal level in many cell types and FDR-significant in excitatory neurons) with no remarkable changes in *FKBP4*, which together would translate to reduced translocation of the GR complex to the nucleus. Moreover, gene expression of the proteins that stabilize the complex^44^ to, i.e. Hsp90 and p23, or facilitate the functional folding of the receptor, i.e. Hsp40 and Hsp70, were generally downregulated, potentially further contributing to the reduction of GR as we showed before in dlPFC^19,36^. Notably, these coordinated trends were less apparent in the bulk, consistent with averaging across heterogeneous cell populations partially obscuring cell-type-specific patterns.

Building on the genetic enrichment results above, we next asked whether cell-type-specific genetic risk converges with cell-type-specific transcriptional dysregulation by integrating SMR with our DGE results. Using single cell cis-eQTLs calculated in 1,047 cortical samples^45^ and the same MDD GWAS^22^, SMR identified between 6 and 172 significant eGenes per cell type (padj < 0.05, HEIDI p > 0.05; **Sup Table 9**), with the largest eGene sets in excitatory (n = 172) and inhibitory (n = 121) neurons, mirroring the cell types most strongly implicated by MAGMA/LDSC. Overlap with nominal cell-type-specific DEGs ranged from 0 to 25 genes per cell type, with the largest overlaps in excitatory neurons (25 genes, 60% concordant direction) and inhibitory neurons (17 genes, 41% concordant direction) (Fig. 4C). Statistical enrichment testing (FET and permutation testing) indicated that none of the observed overlaps exceeded chance expectation (all permutation P > 0.43), similar observation to the non-significant SMR-DGE overlap at the bulk level. Despite the absence of global enrichment, several overlapping genes stood out as biologically convergent. *EPHA7* in astrocytes was concordantly upregulated in both SMR and DGE (b_SMR = 0.059, logFC = 0.37), reinforcing ephrin-Eph signaling as a recurring convergent signal across genetic and transcriptomic layers. *MERTK*, regulating microglial and astrocytic phagocytic clearance of synaptic debris, showed the largest concordant effect size in excitatory neurons (b_SMR = 0.021, logFC = 0.570), and its upregulation has recently been implicated in excitatory synapse loss in mouse models of early life adversity^46^. *CACNA2D1*, encoding a voltage-gated calcium channel subunit, was concordantly downregulated in inhibitory neurons, extending the calcium-signaling dysregulation also implicated among the shared cross-cell-type DEGs.

We next compared each cell-type-specific DGE profile to the bulk-tissue MDD signatures from the aggregated, FC-aggregated model above. Genome-wide pairwise correlation of logFC-ranked differential expression profiles across shared genes revealed overall limited concordance between bulk-tissue and single-cell signatures, with the highest correlations observed in microglia (*ρ* = 0.15) and OPCs (*ρ* = 0.13). When the analysis was restricted to genes nominally dysregulated in both datasets, concordance increased across several non-neuronal cell-types, with the strongest overlap observed in endothelial cells (*ρ* = 0.36), OPCs (*ρ* = 0.28), oligodendrocytes (*ρ* = 0.27), microglia (*ρ* = 0.26), and fibroblasts (*ρ* = 0.26). Together, these findings suggest that the bulk-tissue MDD signature reflects a composite signal that is distributed across multiple non-neuronal cell populations rather than strongly driven by a single cell-type (RRHO plots can be found in: **Fig. S5A)**.

To identify coordinated transcriptional programs altered in MDD, we performed GSEA across all major cell classes (Fig. 4D, **Sup Table 10)**. Neuronal populations exhibited a noteworthy asymmetry: excitatory neurons showed broad negative enrichment of synaptic organization, dendritic spine morphogenesis, axonogenesis, and calcineurin-mediated signaling, with minimal compensatory positive enrichment, consistent with impaired excitatory synaptic and structural plasticity seen in neurodevelopmental disorders^47^. Inhibitory neurons, by contrast, were characterized by enrichment of RNA processing, splicing, vesicle transport, and steroid hormone receptor signaling, with only modest immune-related pathway depletion, suggesting an active post-transcriptional adaptive response. Among glial populations, astrocytes showed downregulation of astrocyte differentiation, cell-matrix adhesion, insulin receptor signaling, and amyloid-beta response programs, alongside enrichment of mitochondrial and ribosomal terms, suggesting altered astrocytic state with metabolic compensation. OPCs and oligodendrocytes similarly depleted synaptic and ECM pathways, with OPCs additionally showing loss of endothelial barrier terms and enrichment of apoptosis-related pathways. Immune and perivascular populations revealed two convergent cross-cell-type themes. Microglia and macrophages showed depletion of canonical immune programs, i.e., NF-κB, Wnt, myeloid differentiation, and leukocyte activation, paired with enrichment of oxidative phosphorylation and mitochondrial terms, consistent with a transcriptional shift away from canonical inflammatory activation. Among perivascular cells, pericytes and fibroblasts converged on depletion of blood-brain barrier, angiogenesis, and endothelial cell programs, pointing to coordinated disruption of neurovascular unit integrity.

To systematically evaluate the relationships across all sub-analyses, we performed genome-wide correlation analyses of differential expression signatures, including sex-stratified (male, female), childhood trauma (CT), and primary diagnosis-specific contrasts (all DGE results can be found in **Sup Table 11**). Across most comparisons, we observed moderate to high concordance, particularly between generalized MDD (gDx) and MDD-only (ρ > 0.76) and PTSD-comorbid (ρ > 0.55) signatures. Similarly, gDx showed consistent agreement with both sex-stratified models (males: ρ > 0.67; females: ρ > 0.6, except for Fibro, ρ = 0.38) and CT-specific analyses (ρ > 0.6), indicating that the generalized disease signal is largely preserved across analytical frameworks. In contrast, comparisons between MDD signatures in males and females revealed weaker concordance, mirroring patterns observed in the bulk-tissue analysis (**Fig. S5B).** Neuronal populations showed moderate high agreement (ρ ~ 0.5), whereas non-neuronal cell-types exhibited low to moderate correlations (ranging from − 0.11 in T cells to ~ 0.3 in astrocytes), suggesting increased heterogeneity in sex-specific effects at the cellular level.

Strikingly, comparisons between primary MDD and PTSD-comorbid signatures showed markedly reduced concordance across cell-types, in sharp contrast to the strong agreement observed in bulk-tissue tissue (**Fig. S5C).** This reduction was most pronounced in neuronal populations, where correlations were weak or even negative (e.g., excitatory subclasses ρ = 0.23; inhibitory subclasses ρ = −0.37). Non-neuronal populations showed more variable but generally modest agreement (e.g., oligodendrocytes and fibroblasts ranging from ρ = 0.18–0.54). To assess whether this divergence persisted at higher levels of biological organization, we next compared pathway-level enrichment profiles using gene set enrichment analysis. Pathway-level correlations between MDD-only and PTSD-comorbid were partially restored in several cell-types, particularly within microglial and macrophage populations (ρ ~ 0.7–0.8), whereas neuronal populations continued to show minimal concordance (ρ ≈ 0). In contrast, gDx-derived signatures remained consistently well correlated with both MDD and PTSD across cell-types at the pathway level.

Altogether, the single cell analysis revealed a compartment-specific transcriptional disruption in MDD. The largest gene-level signal was concentrated in excitatory and inhibitory neurons, with converging evidence for impaired synaptic scaffolding, GABAergic circuit integrity, and stress-axis dysregulation. Non-neuronal populations, while showing more modest gene-level changes, collectively accounted for most of the concordance with the bulk-tissue MDD signature, implicating glial and perivascular components as major contributors to the cortical pathophysiology captured in bulk-tissue. Pathway-level analyses further revealed cell-type-specific adaptive responses, including metabolic compensation in astrocytes and immune program depletion in microglia, along with disruption of neurovascular unit integrity across perivascular populations. Critically, while the generalized disease signal was robust across diagnostic strata and sex at the pathway level, gene expression signatures were highly divergent between MDD-only and PTSD-comorbid conditions highlighting a level of diagnostic specificity that was concealed in bulk-tissue transcriptomic analyses.

### Cell-cell interaction across the cortical environment

The functional annotation so far suggested mechanisms like neuroglia synaptic processes, neuro-oligo axonal guidance, vascular interactions and immune signaling to hold a key role in MDD. We reasoned that cellular communications across the major cell-types could be therefore altered in MDD. We employed the CellChat algorithm to infer ligand-receptor interactions within and across cell-types in the MDD subgroup and compare them to controls. We intersected 1,436 signaling genes across our dataset and the CellChatDB which matched to 70 pathways.

Quantification of intra- and intercellular communication within the neuroglia-vascular cortical microenvironment revealed an overall reduction in both the number and strength of inferred interactions in MDD relative to controls (Fig. 4E–G). Detailed evaluation of interaction patterns (**Fig. S6A**) showed increased autocrine signaling in endothelial cells (+ 15), excitatory neurons (+ 6), mural cells (+ 4), oligodendrocytes (+ 2), and astrocytes (+ 1), whereas reduced autocrine activity was observed in inhibitory neurons (−23), astrocytes (−22), microglia (−10), macrophages (−15), fibroblasts (−13), T cells (−12), and OPCs (−2). Paracrine interactions were overall decreased, but the enriched ones in MDD were predominantly directed toward astrocytes, including signaling from mural cells (+ 10), endothelial cells (+ 9), fibroblasts (+ 6), and oligodendrocytes (+ 3). Additional increases were observed in signaling pathways involving endothelial cells, including mural-to-endothelial (+ 8), excitatory-to-endothelial (+ 7), fibroblast-to-endothelial (+ 6), and OPC-to-endothelial (+ 3) interactions. While overall all the incoming and outgoing interactions across cells were decreased in MDD, the most pronounced decrease was observed in immune cell populations, which showed 1,068 fewer inferred interactions relative to controls (permutation test, 10,000 iterations, P < 1 × 10^−4^) (**Fig. S6B).**

We then annotated these communications to the respective 70 active signaling pathways (Fig. 4G, **Fig. S6C**), which revealed a marked decrease of intercellular signaling networks in MDD relative to controls. Pathways that were control-enriched (n = 48) were linked to broad neuronal and interactions, including glutamatergic and GABAergic signaling, RELN, NCAM, APP, and multiple axon-guidance pathways (EPHA/B, SLIT, UNC5, Netrin, SEMA4/6), together with ECM and angiogenic signaling modules (VEGF, ANGPT, PDGF, TGFB, laminin, collagen, agrin). In contrast, MDD was characterized by enrichment of a small subset of pathways (n = 4) linked to endothelial junctional signaling (JAM, PECAM1), myelin-associated communication (MAG), and structural guidance signaling (SEMA3), suggesting a shift from broad homeostatic cortical communication toward a more limited vascular and remodeling-associated signaling state. Functional similarity analysis revealed a marked reorganization of intercellular signaling architecture in MDD, particularly involving the EPHA, UNC5, and SEMA3 pathways (Fig. 4H). Whereas EPHA clustered with synaptic adhesion and neuronal communication pathways in controls, in MDD it shifted into a distinct module enriched for axon guidance and immune-associated signaling programs, with UNC5 and SEMA3 exhibiting analogous changes in their functional relationships. These findings suggest disease-related rewiring of guidance cue-mediated cellular communication.

The notably low correlation between the single cell transcriptomic profiles of the MDD-only and the PTSD comorbid subgroups prompted us to explore whether the inter- and intracellular interactions similarly reflected this heterogeneity. Interestingly we observed that the global disease CellChat signature was driven primarily by the PTSD-comorbid subgroup (**Fig. S6G-I**), whereas MDD-only (**Fig. S6D-F**) exhibited a partially distinct and in several pathways opposing communication architecture to both the overall disease signature and the PTSD-comorbid subgroup. Despite these differences, the two diagnostic subgroups showed concordant inactivation of pathways linked to synaptic function and cell adhesion/axon-guidance (NRXN, EPHA, Netrin, GABA-A/B, NRG) revealing a shared stress-related disorder foundation of dysregulated communication pathways. In contrast, the discordantly activated pathways in PTSD-comorbid vs the MDD-only subgroups indicated that PTSD comorbidity preferentially alters singling pathways associated with neuroplasticity (Glutamate, PTPR, CNTN, NCAM, SLITRK, CADM), axon guidance (SLIT, UNC5, FLRT), vascular support and ECM signaling (APP, LAMININ, PTN, SEMA6, FGF, JAM), and cholesterol-related signaling. Together, these findings suggest that PTSD comorbidity adds a distinct communication profile characterized by disrupted excitatory, guidance, and neurovascular signaling, complementing a shared MDD-associated core of synaptic dysfunction.

Overall, CellChat analysis revealed a fundamental reorganization of cortical intercellular communication in MDD, characterized by a global reduction in interaction number and strength, represented with a shift from homeostatic signaling toward a restricted state dominated by endothelial junctional and myelin-associated signaling, suggesting broad deterioration of the neuroglia-vascular communication network.

### Ephrin signaling pathway dysregulation in MDD

Across the various analyses performed throughout this study, the transcriptomic and genetic evidence seems to highlight a significant dysregulation of the Ephrin signaling pathway in MDD as well as a generalized dysregulated stress response. Our previous work has shown that dexamethasone (DEX) induces a robust stress response in iPSC-derived neurons, closely recapitulating the transcriptional patterns observed in the postmortem cortex of individuals with stress-related psychiatric disorders^36^. Among the genes with significantly upregulated expression patterns upon treatment with glucocorticoids was *EPHA2* (logFC = 1.34, p.adj = 6.8e-04). From the current study, supporting the genetic basis, the SMR analysis identified *EPHA7* gene as an eGene (*p.adj* = 0.006, pHEIDI = 0.07), with a positive effect estimate (b = 0.08), suggesting that genetically regulated EPHA7 expression may contribute to MDD risk.

Across the bulk-tissue transcriptomic analyses (and subanalyses), Ephrin receptor signaling also emerged as a consistently dysregulated pathway. The strongest and most robust signal was observed for *EPHA2*, which showed significant upregulation across all FC-aggregated (sub)analyses, including the main MDD analysis, sex-stratified MDD analyses, and disorder-specific ones (logFC range: 0.26–0.84, FDR < 10^−3^; Fig. 5A). This convergence across datasets and modelling strategies supports EPHA2 as a core and highly reproducible molecular feature of disease-associated transcriptional dysregulation in the bulk-tissue analysis. Conversely, *EPHA3* was consistently downregulated across analyses (main MDD analysis, female MDD analysis, and PTSD comorbid), indicating that Eph receptor signaling dysregulation is not uniform but instead involves bidirectional regulation of receptor subtypes (**Sup Table 8–9**). At the ligand level, *EFNA1* was detected across most (sub-)analyses to be modestly upregulated (**Sup Table 8–9**). These findings suggest that Ephrin signaling alterations in stress-related psychopathology are characterized by robust EPHA2-driven activation alongside subtype-specific and directionally opposing regulation of additional Eph receptors.

At single-cell resolution, FDR-significant dysregulation of ephrin pathway genes was observed across distinct cellular compartments, supporting cell-type-specific perturbation of Eph/ephrin signaling in MDD. In the main MDD comparison (gDx), *EPHA8* was significantly upregulated in excitatory neurons (logFC = 0.43, FDR = 0.049), whereas inhibitory neurons showed significant downregulation of *EPHA4* (logFC = −0.28, FDR = 0.005) and *EFNB2* (logFC = −0.39, FDR = 0.007). In parallel, the ephrin ligand *EFNA5* was significantly upregulated across the oligodendrocyte lineage, including both oligodendrocytes (logFC = 0.65, FDR = 0.009) and OPCs (logFC = 0.77, FDR = 0.009). Notably, subgroup analyses further supported dysregulation of ephrin signaling, with the strongest recurrence observed in the PTSD-comorbid subgroup, in which multiple Eph receptors and ligands (including *EFNA5, EPHA4, EPHA8, EPHB1/2/4*) remained significantly altered, whereas the MDD-only subgroup showed a more restricted pattern involving *EPHA4* and *EPHA5*. Together, these findings suggest cell-type-specific remodeling of ephrin-mediated neuron-glia communication, rather than uniform pathway dysregulation across cell-types.

Consistent with these transcriptional changes, CellChat analysis revealed that ephrin-related signaling was among the most consistently reduced and functionally diverse communication pathways in MDD (Fig. 5B), with particularly diminished activity in excitatory neuron-glia and neuron-vascular interactions. This could suggest that ephrin pathway dysregulation may reflect a shift in neuron-glia-vascular communication rather than uniform transcriptional activation or repression of the signaling members.

Apart from the critical role ephrins are known to play in axonal guidance and synaptic plasticity, they significantly contribute to neurovascular signaling and BBB regulation^48^, so we sought to investigate whether these observed changes would be captured in the blood of individuals with MDD. We analyzed 1,463 plasma proteins from 54,219 UKBB subjects with varying depression diagnoses and detected a consistent pattern of upregulation of ephrin receptors and ligands across individuals with depression, irrespective of the diagnostic tool used to register the diagnosis (Fig. 5C).

Finally, the triad of mechanisms controlled by ephrins are also found in Alzheimer’s disease related dementias (ADRD), where demyelination, synaptic plasticity and BBB leakiness are hallmarks of the disease^49–52^. We thus set to compare whether the cortical changes in ephrin-related signaling molecules could be comparable, signifying a common mechanism in the two diseases (Fig. 5D). We utilized the publicly available data from Mathys et al.^53^, focusing only on the PFC region. This analysis revealed a clear cell-type specific structure of Ephrin signaling across disorders, with non-neuronal cell-types, particularly astrocytes and perivascular cells exhibiting strong concordance in transcriptional directionality between AD and MDD, suggesting a conserved glial and vascular component of Ephrin dysregulation. In contrast, neuronal populations showed heterogeneity, with excitatory neurons showing partial concordance (75% same direction), whereas inhibitory neurons exhibited a pronounced discordance (27% same direction), indicating divergent regulatory control of Ephrin signaling within inhibitory circuitry across the two disorders.

A summary of the most important results that show a unified stress response spanning neuroglia, neurovascular and neuro-immune interactions across analysis can be found in Fig. 5E. Together, these multi-scale data in the human FC -- from bulk-tissue tissue and cell-type specific genetic regulation of genetic risk to the respective expression alterations and intercellular signaling -- converge on a robust and spatially structured dysregulation of ephrin signaling in MDD, implicating it as a process involved in disease-associated axonal guidance, and BBB permeability that together could be detrimental in neuronal/synaptic plasticity.

## Discussion

Our study is the first to analyze new and published postmortem brain transcriptomics datasets to establish an FC-aggregated mRNA expression profile of MDD at both bulk-tissue and single-cell resolution with the goal to define a unified molecular architecture of MDD across FC. Across analytical frameworks in the bulk-tissue level, we identify a conserved transcriptional program that is shared across cortical regions and robust to clinical heterogeneity. At the single-cell level, we observed disrupted transcriptional and cellular communication networks, with compromised neuroglial and neurovascular interactions, particularly in pathways regulating vascular integrity and axonal guidance. Importantly, these findings extend beyond neuron-centric models, implicating coordinated alterations across glial, immune, and vascular components, with ephrin signaling emerging as a convergent molecular substrate linking these disruptions.

Through the bulk-tissue RNA sequencing analysis we detected common dysregulated profiles in three different FC subregions, suggesting that a stable core molecular signature of MDD is recoverable from bulk-tissue FC tissue regardless of subregional sampling. This extends earlier region-specific observations^12,18,19,36,54^ and demonstrates that aggregation across subregions amplifies power to detect weaker, distributed signals, while preserving biologically meaningful regional specificity. Among the most consistently downregulated genes were canonical somatostatin interneuron markers, SST, CORT, and DLX6-AS1, replicating and reinforcing the GABAergic interneuron hypothesis of MDD^55–57^. Alongside these neuronal deficits, the upregulation of immune-related genes (*IL1RL1, SERPINA3*) and genes linked to ECM and vascular biology (*CLU, EMP3, EPHA2*) suggest that the bulk-tissue MDD signal integrates concurrent neuronal dysfunction and non-neuronal activation.

This was further reinforced by the spatial registration of the MDD signal onto the cortical column which showed enrichment in leptomeningeal space and Layer 1, layers rich in astrocytic endfeet and harboring meningeal fibroblasts, endothelial cells, and border-associated macrophages, alongside enrichment in deep WM. These spatial patterns converged with the pathway analysis, where positively enriched GO terms were dominated by ECM remodeling, immune regulation, vascular cell biology, and TOR signaling, rather than classical neuronal processes. Taken together, the spatial and functional data suggested that the bulk-tissue MDD signal is disproportionately driven by non-neuronal, laminar-boundary cell populations. This interpretation was directly validated by single-cell correlation analyses, in which non-neuronal cell-types, oligodendrocytes, microglia, OPCs, endothelial cells, and fibroblasts, showed higher concordance with the bulk-tissue signature than the neuronal populations. This finding aligns with a growing body of work showing that glial and vascular dysfunction may be primary, rather than secondary, features of cortical pathology in neuropsychiatric disorders^58–60^.

At single-cell resolution, following the aggregated model we established through the bulk-tissue analysis, we also saw increased DEG discoverability across cell-types with the increasing number of nuclei collected here. Excitatory neurons harbored the largest number of DEGs, with broad negative enrichment of synaptic organization, dendritic spine morphogenesis, axonogenesis, and calcineurin-mediated signaling, collectively indicating impaired excitatory synaptic and structural plasticity. The upregulation of *SEMA3A*, an axon guidance gene, and downregulation of *PDGFD*, which supports vascular and glial cells, further suggests altered intercellular signaling capacity. Notably, excitatory neurons also showed upregulation of *FKBP5*, a stress-responsive co-chaperone and negative regulator of GR, consistent with its well-established role in HPA axis dysregulation in MDD^41,61^. This single-gene observation prompted a broader examination of the GR signaling machinery across cell-types, revealing a coordinated disruption: FKBP5 upregulation was accompanied by downregulation of the GR-stabilizing chaperones HSP90, HSP70, HSP40, and p23, as well as downregulation of NR3C1 itself, suggesting that chronic glucocorticoid dysregulation impairs GR complex assembly and nuclear translocation rather than representing an isolated stress-response gene change. This pattern converges with the enrichment of corticosteroid response pathways in MOFA Factor 4 and with our prior iPSC-based work demonstrating that molecular glucocorticoid stress response recapitulates postmortem cortical transcriptional signatures^36^, together suggesting that HPA axis dysregulation leaves a durable, multi-cellular transcriptomic imprint across the MDD cortex. In inhibitory neurons, the downregulation of *DLX6-AS1, EPHA4*, and *TRPC3* aligns with the bulk-level depletion of inhibitory markers. Together, these neuronal findings are consistent with the Excitatory/Inhibitory imbalance hypothesis of MDD^62^ but provide cell-type-level resolution that locates the primary deficit in excitatory synaptic scaffold remodeling rather than interneuron cell loss per se.

Among non-neuronal populations, the oligodendrocyte lineage exhibited disruption of ECM and myelination-associated programs. The convergent upregulation of *EFNA5* in both OPCs and oligodendrocytes, paired with downregulation of its receptor *EPHA4* in inhibitory neurons, defines a reciprocal neuron-glia ephrin-Eph signaling disruption that may impair activity-dependent myelination, an increasingly recognized mechanism linking neural activity to WM plasticity^63^. Astrocytes showed dysregulation of ECM adhesion, neurotrophic receptor trafficking (*SORCS1*), and somatostatin receptor signaling (*SSTR2*), the latter potentially reflecting a compensatory upregulation in the context of documented somatostatin interneuron deficits. In the perivascular microenvironment, fibroblasts showed downregulation of ECM and cytoskeletal genes (*LAMA2, CALD1, ARHGAP42*), while mural cells exhibited changes consistent with altered survival signaling (*BCL2L2, ADGRB2*). The spatial enrichment of the bulk-tissue MDD signal in the leptomeningeal space, combined with these perivascular transcriptomic alterations, suggests that the meningeal-perivascular niche is a site of active structural and signaling remodeling in MDD, a compartment that has only recently gained attention in neuropsychiatric disease^17,64,65^. Notably, the concordance of several perivascular DEGs, i.e., *LAMA2, ARHGAP42*, with the independent dataset from Hwang et al.^12^ strengthens confidence in these findings.

Although our bulk cortical DGE signatures showed little overall enrichment for loci implicated by psychiatric GWAS and no significant excess overlap with SMR-prioritized genes, these findings are consistent with growing evidence that common variant liability and disease-state transcriptional alterations often capture distinct layers of disease biology. Genetic risk variants primarily reflect inherited susceptibility mechanisms operating across development and cellular homeostasis, whereas differential expression in postmortem cortex may additionally incorporate the cumulative effects of illness progression, environmental exposures, stress, treatment history, and compensatory responses. Nevertheless, the convergence observed among a subset of genes across DGE, SMR, and TWAS highlights biologically meaningful pathways that may bridge genetic risk and molecular pathology in depression. Notably, genes such as *NCOA1* and *PBRM1* implicate glucocorticoid receptor signaling and chromatin remodeling, linking inherited susceptibility to stress-response mechanisms that emerged throughout our transcriptomic analyses. Similarly, *CTTNBP2* and *NCKIPSD* support disruption of synaptic organization and dendritic spine maintenance, while *TRIM8*, *SPATA2*, *RELT*, and *USP24* point to immune-regulatory and ubiquitin-proteasome pathways increasingly implicated in mood disorders. The strong concordance between SMR and TWAS signals, contrasted with the modest overlap between genetically informed genes and observed differential expression, further suggests that genetically regulated expression represents a more stable component of disease susceptibility than the broader transcriptomic alterations observed in affected brain tissue. Together, these findings support a model in which depression risk is concentrated within neuronal and oligodendroglial genetic programs, while downstream disease-state transcriptional changes extend to additional stress-responsive, inflammatory, and circuit-level processes that are only partially captured by inherited genetic liability.

The cell-type-specific analyses further refined this framework by demonstrating that genetic liability for both MDD and PTSD is concentrated primarily within excitatory neurons, inhibitory neurons, and oligodendrocyte precursor cells. The remarkable concordance between MAGMA and S-LDSC provides strong evidence that neuronal and OPC regulatory programs represent core cellular substrates of psychiatric disease risk. In contrast, astrocytic enrichment was comparatively modest and endothelial or immune cell populations showed little evidence of genetic enrichment despite exhibiting prominent transcriptional alterations in our expression analyses. These findings suggest that many neuroinflammatory and vascular signatures observed in disease tissue may arise secondarily to disease processes, stress exposure, or environmental influences rather than reflecting primary mechanisms of inherited risk. Consistent with the bulk-tissue results, integration of cell-type-specific DGE and SMR data revealed limited global enrichment beyond chance expectations, underscoring the complex relationship between genetic susceptibility and disease-associated gene expression changes. However, several convergent genes, including EPHA7, MERTK, and CACNA2D1, emerged as biologically compelling candidates linking genetic variation to cell-type-specific pathology. Collectively, these results support a model in which genetic risk preferentially targets neuronal and oligodendroglial regulatory networks, while downstream transcriptional adaptations propagate across multiple interacting cellular populations within the depressed cortex.

Cell-cell communication inference revealed a global reduction in both interaction number and strength in MDD, with the most pronounced loss in immune cell populations. The interactions primarily active in controls were mainly canonical neuronal communication pathways, glutamatergic and GABAergic signaling, RELN, NCAM, and multiple axon guidance modules, along with ECM and angiogenic signaling (VEGF, ANGPT, PDGF, TGFB, collagen). In contrast, MDD was characterized by activation of only four pathway categories, linked to endothelial junctional integrity (JAM, PECAM1), myelination-associated communication (MAG), and structural guidance (SEMA3). This shift, from a diverse, neuronally-oriented communication network to a restricted vascular-remodeling state, represents a potential reorganization of cortical circuit architecture in depression. Notably, the increased communications from perivascular and oligodendrocyte sources towards astrocytes may reflect compensatory recruitment of astrocytic support in the context of deteriorating neurovascular unit function. The disease-related rewiring of ephrin signaling architecture, moving from a synaptic adhesion module in controls to an axon guidance and immune signaling cluster in MDD, further substantiates the emergence of ephrin signaling as a cross-cellular disease axis.

Across all analytical modalities, ephrin-Eph signaling emerged as the most consistently dysregulated pathway in MDD, supported by convergent evidence spanning genetic, transcriptomic, cellular, communication, and proteomic layers. Ephrin ligands and their Eph receptors are integral to neurodevelopmental processes, including axon guidance, synaptic formation, and the establishment of topographic neural maps^66^. Beyond development, the Eph/ephrin system has been implicated in neuropsychiatric disorders. Studies have shown that proteolytic cleavage of Eph receptors, like EphB2 by neuropsin in the mouse amygdala, can modulate anxiety-related behaviors, highlighting the pathway’s relevance to emotional regulation^67^. Additional molecules that cleave the Eph receptors like metalloproteases (ADAM, MMP) have been recently linked to increased stress susceptibility and depressive-like behavior in mouse models^68^ and intergenerational transmission of traumatic stress^69^. While activation of the EPHB receptors promotes synaptic plasticity^70^, activation of the EPHA receptors leads to dendritic spine elimination^51^, thereby impairing it. Moreover, EPHA2 receptor activation, as observed in this study both in the cortex and blood, can occur after brain injury and contribute to inflammation by promoting BBB leakage^71^. Notably, the biological processes regulated by ephrin-Eph signaling are also disrupted in ADRD. Consistent with this overlap, we observed strong concordance of ephrin-related transcriptional changes in astrocytes and vascular-associated cell types between MDD and AD, suggesting that glial and neurovascular ephrin signaling may represent a conserved mechanism across psychiatric and neurodegenerative disorders. Recent work further implicates ephrin signaling in astrocyte- and microglia-mediated neuroinflammation in AD while demonstrating preclinical therapeutic benefit from targeting this pathway, raising the possibility that similar approaches could be leveraged to modulate neuroinflammatory processes in MDD^72^. Together with our observation that EPHA7 genetic variation is associated with MDD risk and that ephrin signaling is consistently dysregulated across genetic, transcriptomic, proteomic, and cellular communication analyses, our findings identify bidirectional Eph/ephrin signaling as a candidate mechanism linking stress-related pathology to disrupted neurovascular and neuroglial communication. These results support further investigation of cell type-specific Eph/ephrin signaling as a potential therapeutic target for restoring intercellular communication in MDD.

A striking finding from our single cell analysis was the dramatic divergence between primary MDD and PTSD-comorbid transcriptional signatures, in contrast to their strong concordance in bulk-tissue tissue which has repeatedly been reported^12,61^. This dissociation was most pronounced in neuronal populations, where correlations between the two diagnostic groups were weakly positive or negative, whereas non-neuronal populations showed modest but more variable correlation. This pattern has important implications for translational work: bulk-based biomarkers may fail to distinguish MDD from PTSD-comorbid presentations, whereas cell-type–specific signatures, particularly from neuronal populations, may provide the optimal resolution for diagnostic stratification and separation of the phenotypes. Furthermore, the finding that the global CellChat disease signature was driven primarily by the PTSD-comorbid subgroup suggests that trauma-related psychopathology adds a distinct, amplified communication-network disruption on top of a shared stress-related substrate.

While this study offers a comprehensive cross-regional and multi-resolution analysis of the MDD transcriptome in the FC, several limitations warrant consideration. Firstly, MDD is a highly comorbid disorder, and our cohorts included individuals with co-occurring PTSD, death by suicideres and psychosis (in order of frequency). Although we ran sensitivity analyses for known confounders, incomplete harmonization of demographic and clinical variables across cohorts may have introduced residual confounding. Moreover, given the substantial clinical and biological overlap between MDD and stress-related psychopathology, some of the molecular signatures identified here may reflect broader stress-response processes rather than MDD-specific effects. Importantly, the predominantly European ancestry composition of our cohorts limits generalizability, and future studies in ancestrally diverse populations will be essential to establish the cross-ethnic robustness of the molecular signatures reported here. The analyzed brain regions were not fully overlapping across individuals, and multi-omic data were not available for every cortical subregion, potentially limiting the completeness of the multi-omic integration. The external validation against Girgenti et al.^18^ was restricted to divergent brain regions, as the two studies share samples, which would have biased a direct correlation analysis on common regions. The single-cell dataset covered dlPFC, mPFC, and oFC, not perfectly overlapping with all three bulk-tissue subregions analyzed, and integration across four independent studies, while carefully harmonized through sequential clustering and filtering prior to integration, may introduce residual batch-related variance that could influence downstream analyses. Additionally, our lower cell-type-specific DEG discoverability rates in comparison to Hwang et al.^12^ could reflect more stringent cell-type annotations and downstream application of linear models on pseudobulked expression. The substantially larger number of DEGs detected in excitatory neurons relative to all other cell-types is in line with the prior studies^12,19^ but also warrants statistical interpretation. Excitatory neurons represent nearly half of all profiled nuclei and are divided into multiple transcriptionally distinct subclasses; the high statistical power conferred by cell abundance, combined with the joint subclass modeling framework, may contribute to inflation of the significant gene count beyond what is attributable to disease-specific biological signal alone. Finally, the causal direction of the observed transcriptomic changes remains inherently unclear in postmortem studies. Medication exposure was not explicitly modeled as a covariate because these data were only available for a subset of donors. Although sensitivity analyses in this subset did not indicate substantial medication-related transcriptional effects, medication-associated influences cannot be definitively excluded. Formal causal mediation analyses to distinguish disease- from treatment-associated effects are likely underpowered in the current cohort and will require larger sample sizes. This is particularly relevant for the ephrin signaling findings, where the convergence of multiple lines of evidence is compelling but mechanistic causality remains to be established. Complementary *in vitro* mechanistic approaches, additional validation at the protein and phosphoproteomic levels, as well as spatially resolved validation approaches will be critical for determining whether the observed transcriptional changes are functional and not compensatory and could inform therapeutic alternatives. Addressing these limitations in future work will be essential for refining the molecular framework of MDD proposed here and for translating these findings toward biological and clinical applications.

This study provides a multi-scale transcriptomic architecture of MDD in the human FC, integrating bulk-tissue regional, single-cell, and multi-omic perspectives to define disease molecular signatures. The data collectively support a model of MDD in which a conserved transcriptional program, robust to regional and clinical heterogeneity but moderated by biological sex and diagnostic subtype, is expressed at the cellular level through coordinated disruption of neuronal synaptic integrity, glial ECM and myelination programs, and perivascular structural maintenance. The convergence of genetic, transcriptomic, proteomic, and functional evidence on ephrin signaling places the Eph-ephrin pathway as a hub of multifaceted cortical dysregulation, linking synaptic remodeling, axonal guidance, and neurovascular function in a unified disease mechanism. These findings reframe MDD from a synapse-centric disorder toward a distributed cortical interface disorder and highlight the perivascular and glial components as mechanistically tractable targets for novel therapeutic development.

## Supplementary Material

Supplementary Files

This is a list of supplementary files associated with this preprint. Click to download.

- Supplementaryinformation.pdf
- SupplementaryTables.xlsx

## Figures and Tables

**Figure 1:**
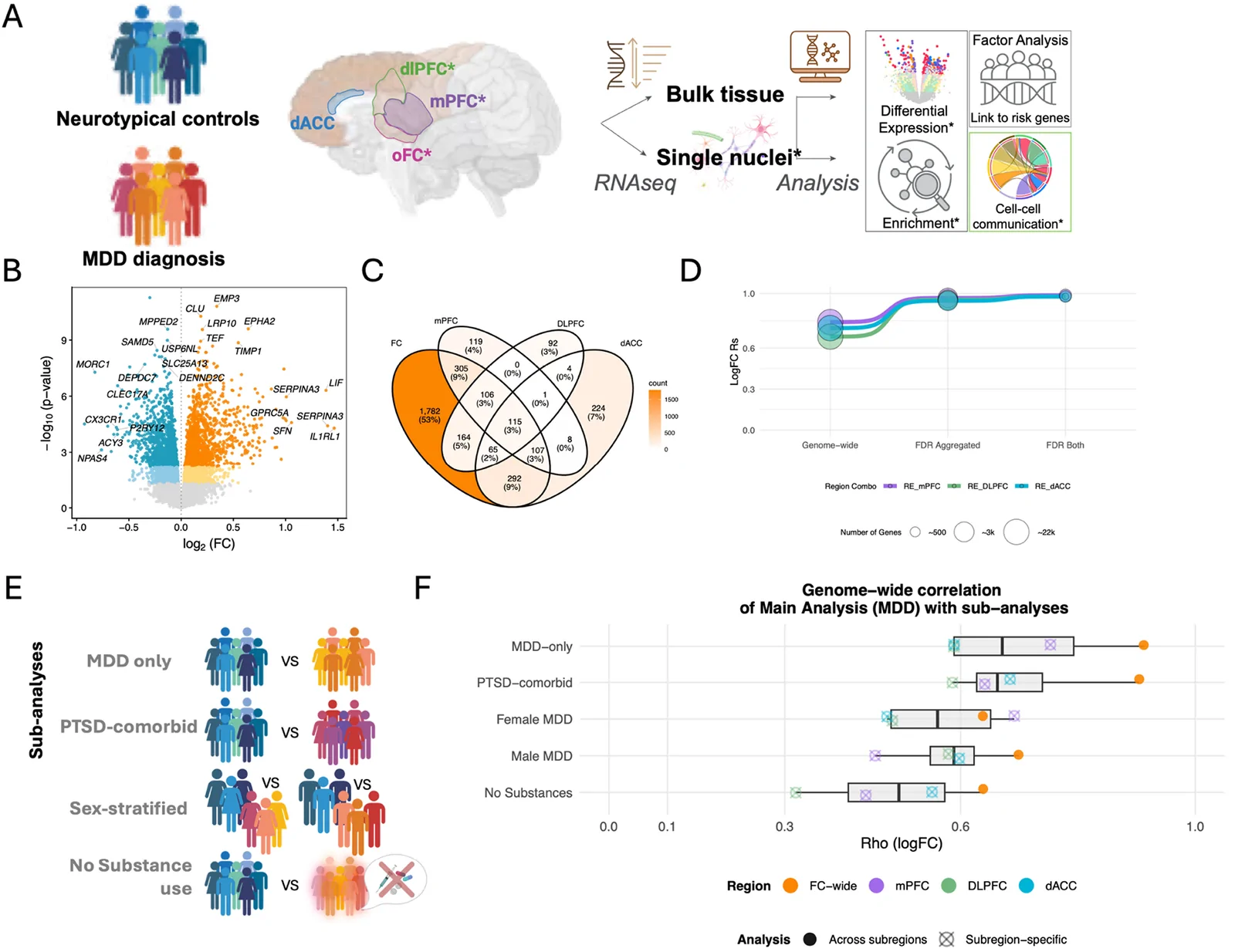
Transcriptomic architecture of MDD across prefrontal cortical subregions. **(A)** Schematic overview of the study design, illustrating bulk RNA-seq profiling across three frontal cortical subregions: medial prefrontal cortex (mPFC), dorsolateral prefrontal cortex (dlPFC), and dorsal anterior cingulate cortex (dACC) — from 286 unique postmortem individuals (138 shared across all three regions), and the analytical frameworks applied. **(B)** Volcano plot of differential gene expression from the FC-aggregated model (N = 286 individuals). Each point represents one gene; the x-axis shows log_2_ fold change and the y-axis shows −log_10_(FDR-adjusted p-value). Genes passing FDR < 0.05 are highlighted (upregulated: red; downregulated: blue); the top 10 most significant genes in each direction are labeled. A total of 2,938 DEGs were identified (1,585 upregulated, 1,353 downregulated). **(C)** Venn diagram showing the overlap of FDR-significant DEGs across the FC-aggregated model and the three region-specific analyses. The majority of region-specific DEGs were recovered in the FC-aggregated model (mPFC: 83%; dlPFC: 82%; dACC: 70%). **(D)** Bump plot displaying Spearman correlations between the FC-aggregated model and each region-specific analysis at three levels of gene selection: genome-wide (all expressed genes), FDR-significant genes from the FC-aggregated model, and genes reaching FDR significance in both the FC-aggregated and the respective region-specific model. Point size reflects the number of shared DEGs contributing to each correlation estimate. Correlations increase substantially as analysis is restricted to more robustly dysregulated gene sets, with near-identical effect size rankings among replicated genes (ρ = 0.98–0.99). **(E)** Schematic overview of the subgroup analyses performed, including stratification by primary diagnosis (MDD-only, PTSD-comorbid), biological sex (male, female), and substance use status (no reported use of any substances). **(F)** Boxplots showing genome-wide Spearman correlations of effect sizes between each subgroup analysis and the primary FC-aggregated model, across the FC-wide and three region-specific models. Each boxplot displays the median (vertical line), interquartile range (box), and whiskers extending to 1.5× the IQR. Correlations were highest for MDD-only (ρ = 0.91) and PTSD-comorbid (ρ = 0.90) subgroups, and lowest for sex-stratified analyses, particularly in female-specific models (ρ = 0.47–0.64), reflecting greater transcriptional divergence by sex than by diagnostic subtype. M: male; F: female.

**Figure 2:**
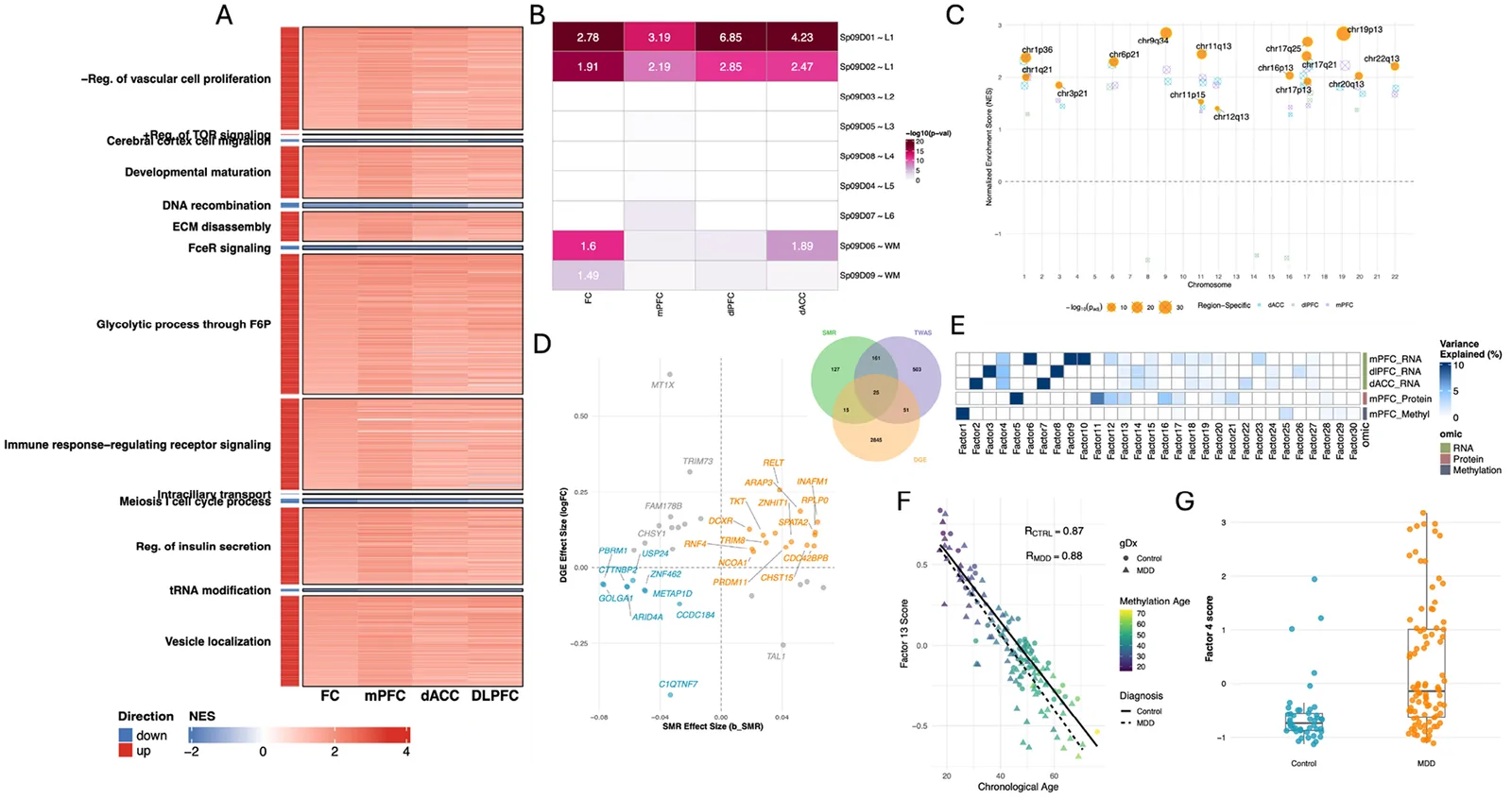
Pathway, spatial, genomic, and multi-omic integration of MDD-associated transcriptional signatures. **(A)** Heatmap of normalized enrichment scores (NES) for the all the significant Gene Ontology Biological Process (GO:BP) pathways in the FC-aggregated model, with corresponding NES values shown for each region-specific analysis (mPFC, dlPFC, dACC). Pathways are grouped along the x-axis into thematic clusters based on semantic similarity. Color reflects NES direction and magnitude (red: positive enrichment; blue: negative enrichment). **(B)** Heatmap displaying spatial enrichment of MDD-associated DEGs across cortical column domains (leptomeninges, layers 1–6, and white matter), estimated by overlap with layer- and domain-specific marker gene sets from a reference single-cell spatial atlas of the human dlPFC. Color reflects level of significance); odds ratios for statistically significant enrichments (FDR < 0.05) are labeled within cells. Consistent enrichment in leptomeninges, Layer 1, and white matter was observed across the FC-aggregated and leptomeninges and L1 in the region-specific models. **(C)** Scatterplot of chromosomal positional enrichment of MDD-associated transcriptional signatures in the FC-aggregated model (orange) and each region-specific analysis (mPFC, dlPFC, dACC; shaded). Each point represents one chromosomal locus; the x-axis shows chromosome number and the y-axis shows NES. Point size reflects −log_10_ of the FDR-adjusted enrichment p-value. The strongest enrichment signals were observed at Chr19p13 (NES = 2.83) and Chr9q34 (NES = 2.85) in the FC-aggregated model, with several loci showing consistent enrichment across all analyses. **(D)** Scatterplot comparing log_2_ fold change effect sizes for genes identified as FDR-significant DEGs (x-axis) and significant SMR eGenes passing HEIDI filtering (HEIDI p > 0.05; y-axis). Points represent genes present in both analyses; concordantly upregulated genes are shown in orange and concordantly downregulated in blue, with discordant genes in gray. Inset Venn diagram shows the overlap of gene sets identified across DEG, SMR, and TWAS analyses, with CTTNBP2 identified at the intersection of all three approaches. **(E)** Heatmap displaying the proportion of variance explained by each of the 30 MOFA latent factors across the multi-omic and multi-regional input views (bulk RNA-seq from mPFC, dlPFC, and dACC; proteomics and DNA methylation from mPFC), computed across the 138 individuals with data available across all modalities. Factors with high R^2^ in a single view reflect omic-specific structure (Factors 1, 5, 2/3/6), while multi-view factors reflect cross-omic or cross-regional variance (Factor 17: multi-omic; Factors 4/15: cross-regional RNA; Factor 13: global). **(F)** Scatterplot of MOFA Factor 13 scores as a function of chronological age in neurotypical controls (circles) and individuals with MDD (triangles). Point color reflects Horvath DNA methylation-based biological age. Solid and dashed regression lines represent fitted slopes in controls and MDD, respectively. **(G)** Boxplot of MOFA Factor 4 scores in neurotypical controls and individuals with MDD. Each boxplot displays the median (horizontal line), interquartile range (box), and whiskers extending to 1.5× the IQR; individual samples are overlaid as points.

**Figure 3:**
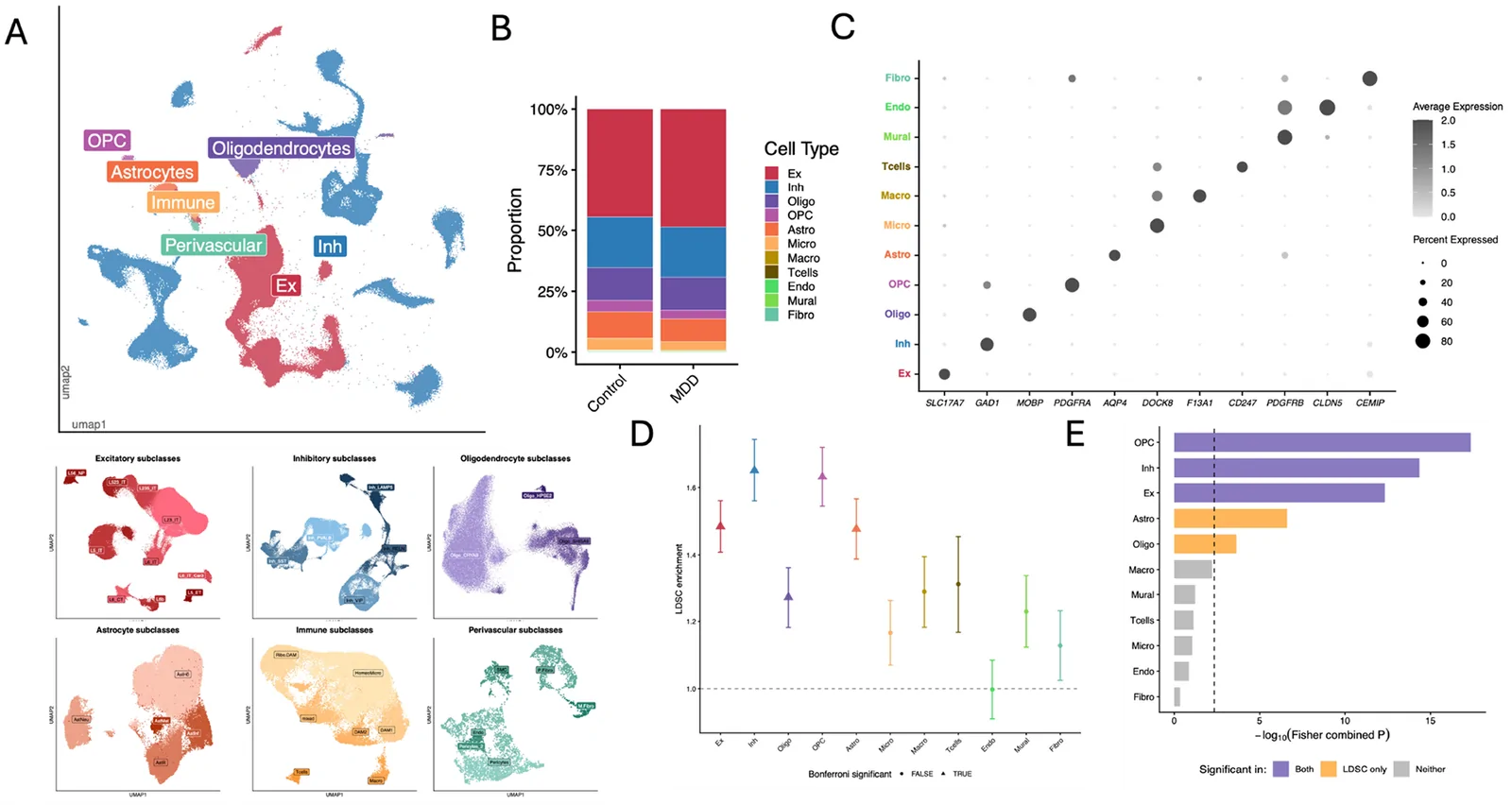
Cell-type annotation and disease-associated genetic enrichment in the human frontal cortex. **(A)** UMAP visualization of 863,511 nuclei integrated across the dlPFC, mPFC, and oFC, colored by annotated major cell class (excitatory neurons, Ex; inhibitory neurons, Inh; oligodendrocytes; astrocytes; oligodendrocyte precursor cells, OPC; immune cells; perivascular cells). Inset UMAPs show subclustering within each major class: excitatory neurons (10 subclasses by cortical layer and projection type), inhibitory neurons (5 subclasses: LAMP5, SST, PVALB, VIP, RELN), oligodendrocytes (3 subclasses), astrocytes (4 subclasses: homeostatic, reactive, inflammatory, neuronal-marker-expressing), immune cells (microglia homeostatic and disease-associated, macrophages, T cells), and perivascular cells (endothelial, mural, fibroblasts). **(B)** Stacked bar plots showing cell-type proportion estimates for each major class in neurotypical controls and MDD samples. After correction for age, sex, ancestry, study, chemistry version, tissue, and donor, no significant cell-type proportion differences survived Bonferroni correction in the MDD vs. control comparison. **(C)** Dot plot of canonical marker gene expression across annotated cell types and subclasses. Dot size reflects the proportion of nuclei expressing the gene within each cell type; color intensity reflects mean normalized expression. Marker genes shown are representative of each major class and selected subclasses, as listed in Supplementary Table 5A–B. **(D)** Dot plot of stratified LD score regression (S-LDSC) enrichment results for MDD genetic risk (PGC MD GWAS, N cases = 412,305) across major cell types, based on cell-type-specific gene expression signatures. The y-axis shows the τ z-score reflecting the conditional contribution of each cell type’s gene set to MDD heritability, after accounting for baseline genomic annotations. Triangles denote cell types reaching FDR < 0.05. Inhibitory neurons (τ z = 3.38, FDR = 8.0×10^−3^) and OPCs (τ z = 2.68, FDR = 0.027) showed significant enrichment, while endothelial cells showed significant depletion (τ z = −2.80, FDR = 0.027), indicating selective contribution of inhibitory neuronal and precursor glial populations to MDD genetic risk. **(E)** Bar plot of combined genetic enrichment across major cell types, ranked by −log_10_(Fisher combined p-value), integrating evidence from MAGMA and S-LDSC. Bar color indicates the source of significance after Bonferroni correction: purple, significant in both MAGMA and S-LDSC; orange, significant in S-LDSC only; grey, not significant in either analysis. Excitatory neurons, inhibitory neurons, and OPCs showed convergent enrichment across both methods, whereas astrocytes and oligodendrocytes were significant only in S-LDSC but exhibited significant combined Fisher p-values.

**Figure 4:**
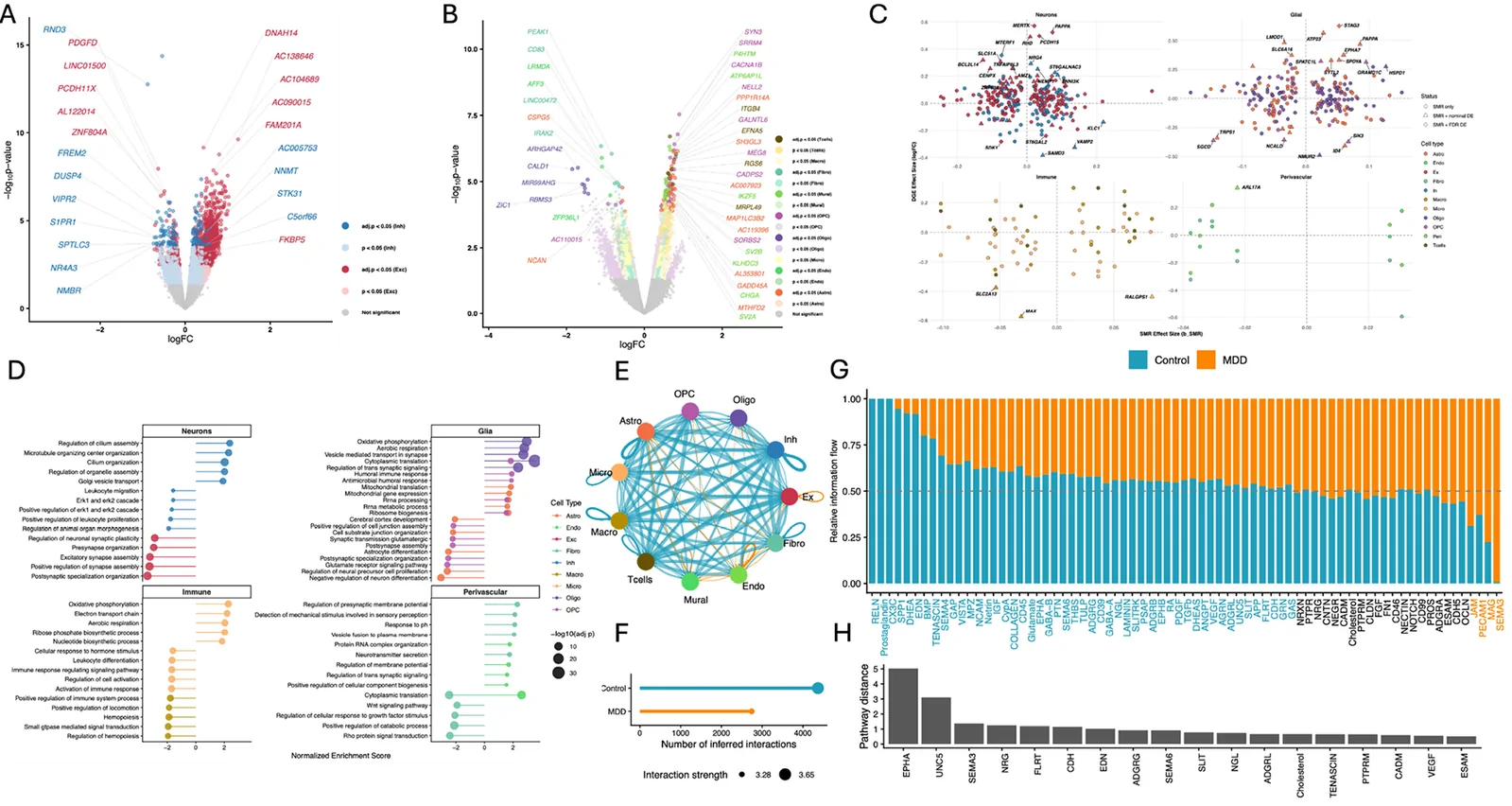
Cell-type-resolved differential expression and cell-cell communication remodeling in MDD. **(A)** Volcano plot of pseudobulk differential expression results for neuronal cell types (excitatory in red; inhibitory in blue). Each point represents one gene in one cell type; the x-axis shows log_2_ fold change and the y-axis shows −log_10_(FDR-adjusted p-value). Bold colors indicate FDR significance (FDR < 0.05), pale colors indicate nominal significance (p < 0.05), and gray indicates non-significant genes. **(B)** Volcano plot of pseudobulk differential expression results for non-neuronal cell types, formatted as in (A). Oligodendrocytes and OPCs are shown in shades of purple, astrocytes in shades of orange, perivascular cells (endothelial, mural, fibroblasts) in shades of green, and immune cells (microglia, macrophages, T cells) in shades of yellow. **(C)** Scatterplots comparing SMR effect sizes (b_SMR; x-axis) and DGE effect sizes (log_2_ fold change; y-axis) for genes identified as significant SMR eGenes within each major cell class: neurons, glial cells, immune cells, and perivascular cells. Points are colored by cell type; point shape reflects differential expression status (circle: SMR significant only; triangle: SMR significant with nominal DE significance, P < 0.05; diamond: SMR significant with FDR-significant DE, adj.P.Val < 0.05). Genes reaching nominal or FDR significance in DE are labeled (up to 10 per cell type, prioritized by combined effect size). **(D)** Lollipop plots displaying GSEA results for each major cell type, showing the top 5 upregulated and top 5 downregulated GO:BP pathways ranked by NES among those reaching FDR significance where available. Each panel represents one cell type group; lollipop color reflects cell types and point size reflects −log_10_(FDR-adjusted p-value). **(E)** Circos plot of inferred cell-cell communication interactions in neurotypical controls (blue) and MDD (orange), estimated using CellChat from aggregated pseudobulk expression profiles. Arc width is proportional to the number of ligand-receptor interactions between each pair of cell types. The most pronounced decreases is in neuron-to-neuron and neuron-to-glia communication. **(F)** Lollipop plot showing the number of inferred significant ligand-receptor interactions for each cell-type pair in controls (blue) and MDD (orange). Point size reflects total interaction strength. **(G)** Stacked bar plots of relative information flow — defined as the aggregate communication probability summed across all ligand-receptor pairs within each signaling pathway — for controls and MDD. Each bar represents one signaling pathway; color indicates the condition in which that pathway shows significantly greater activity (blue: controls; orange: MDD). Pathways with significantly greater information flow in controls include glutamatergic, GABAergic, RELN, and NCAM signaling; pathways with significantly greater information flow in MDD include JAM, PECAM1, MAG, and SEMA3 signaling, reflecting a shift from homeostatic neuronal communication toward endothelial adhesion and myelination-associated pathways. **(H)** Bar plot of functional similarity distance between MDD and control communication profiles for each signaling pathway, computed as the Euclidean distance between condition-specific communication probability vectors across all cell-type sender-receiver pairs within each pathway. Larger distance values indicate pathways whose overall intercellular communication architecture is most divergent between conditions, independent of the direction of change. Ephrin-EphA showed the greatest functional divergence between MDD and controls.

**Figure 5:**
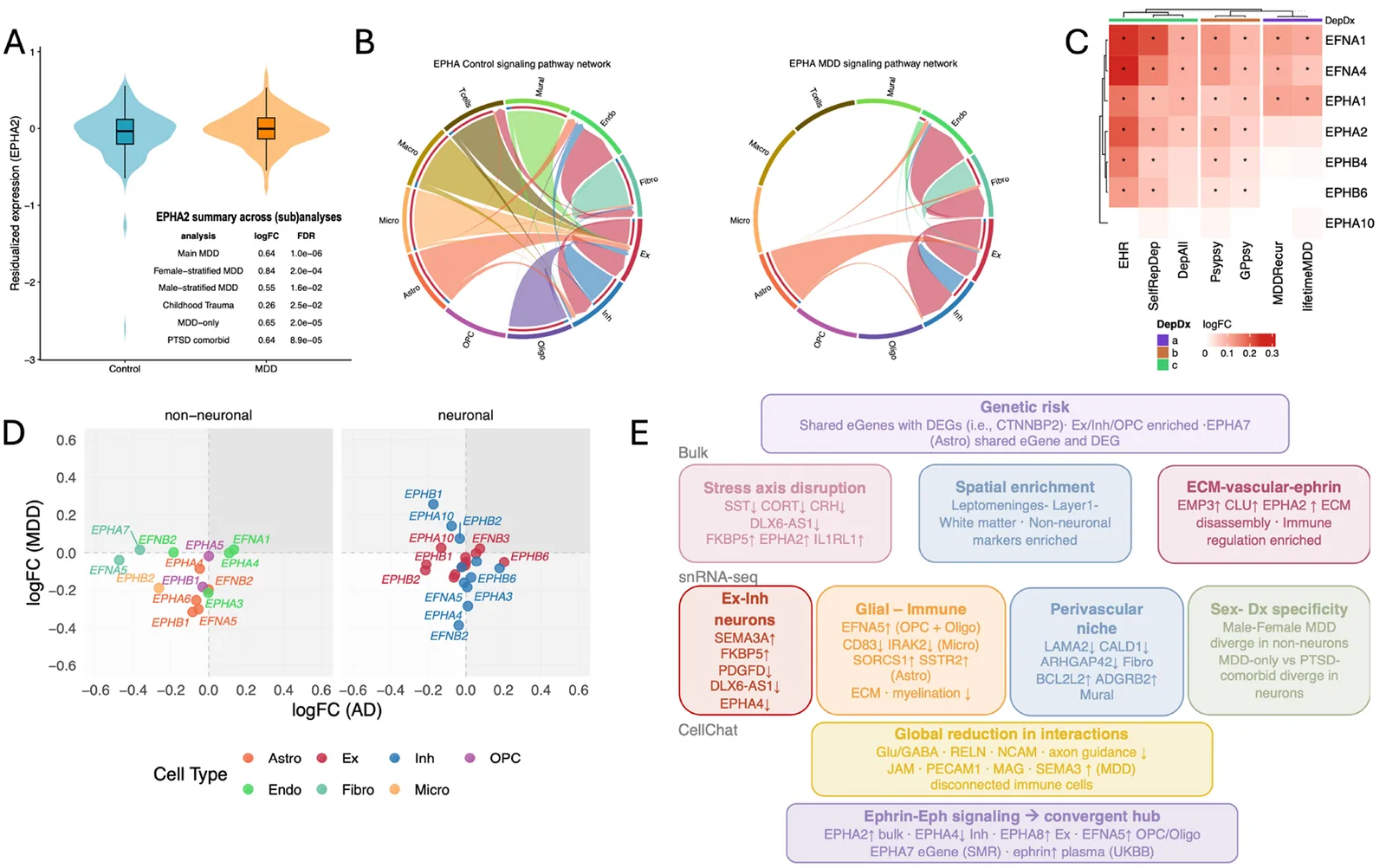
Ephrin-Eph signaling as a convergent hub in MDD across transcriptomic, proteomic, and cross-disorder analyses. **(A)** Violin plot of residualized EPHA2 expression in neurotypical controls and MDD in the FC-aggregated bulk model, after regressing out all model covariates. The embedded bar plot within each violin shows the mean and 95% confidence interval of the residualized expression. Inset summary panel displays log_2_ fold change estimates FDR corrected p values for EPHA2 across all primary and sensitivity analyses (FC-aggregated, sex-stratified, diagnosis-stratified, and childhood trauma models), demonstrating consistent upregulation of EPHA2 in MDD across analytical frameworks. **(B)** Circos plots displaying inferred ligand-receptor interactions within the ephrin signaling pathway in neurotypical controls (left) and MDD (right), derived from CellChat analysis. Arc width is proportional to inferred communication probability between sender (ligand-expressing) and receiver (receptor-expressing) cell types. **(C)** Heatmap of log_2_ fold change in plasma protein levels of ephrin ligands and receptors across seven UK Biobank depression phenotypes of varying diagnostic depth: EHR (ICD-coded depression from electronic health records); SelfRepDep (self-reported depression treatment-seeking); DepAll (presence of low mood or anhedonia, plus GP or psychiatrist contact); GPpsy (having consulted a General Practitioner); Psypsy (having consulted a psychiatrist); MDDRecur (lifetime MDD with recurrence, derived from DSM-5 criteria using PHQ-9 in the Online Mental Health follow-up); and LifetimeMDD (lifetime MDD derived from DSM-5 criteria using PHQ-9, without recurrence requirement). Color reflects log_2_ fold change (white: 0; red: 0.3); asterisks indicate FDR-significant associations (FDR < 0.05). Ephrin protein levels were consistently elevated across multiple depression phenotypes, with strongest effects in the more stringent diagnostic definitions (MDDRecur, LifetimeMDD), suggesting that ephrin dysregulation tracks with diagnostic specificity. **(D)** Scatterplot of differential expression effect sizes for nominally significant ephrin-related genes shared between MDD (this study, y-axis) and Alzheimer’s disease (Mathys et al., 2024, x-axis), matched to the best available corresponding cell type across studies. Points are color-coded by cell type. The plot is divided into non-neuronal cell types (left panel) and neuronal cell types (right panel). Concordantly dysregulated genes (showing the same direction of effect in both MDD and AD) are shown in the upper-right (shared upregulation) and lower-left (shared downregulation) quadrants, while discordant genes occupy the off-diagonal quadrants. **(E)** Summary schematic illustrating the most consistent dysregulation findings in MDD. Together these findings position ephrin-Eph signaling as a convergent hub linking disrupted axonal guidance, synaptic remodeling, and neurovascular integrity in MDD.

## Data Availability

Bulk RNA-seq data and previously published dorsolateral prefrontal cortex (dlPFC) single-nucleus RNA-seq data described in this manuscript are available through the PsychENCODE NDA Collection #5275 (DOI: 10.15154/afnz-8276); data are available for general research use according to consortium requirements for data access and attribution. New single-nucleus RNA-seq data generated from the medial prefrontal cortex (mPFC) as part of this study will be deposited in the NIMH Data Archive (NDA; accession pending) upon publication. Previously published dlPFC single-nucleus RNA-seq data from Daskalakis et al. (2024) are additionally available via Synapse (https://www.synapse.org/Synapse:syn25611941). Previously published dlPFC single-nucleus RNA-seq data from the McGill cohort are available on GEO (Nagy et al. 2020: GSE144136; Maitra et al. 2023: GSE213982). Orbitofrontal cortex (oFC) single-nucleus RNA-seq data are available from Fröhlich et al. (2024) under GEO accession GSE254569. Major depression GWAS summary statistics were obtained from the Psychiatric Genomics Consortium (Adams et al. 2025; available via Figshare: https://figshare.com/articles/dataset/GWAS_summary_statistics_for_major_depression_PGC_MDD2025_/27061255). PTSD GWAS summary statistics were obtained from Nievergelt et al. (2024), schizophrenia GWAS summary statistics from Trubetskoy et al. (2022), and bipolar disorder GWAS summary statistics from O’Connell et al. (2025). Gene expression weights used for TWAS analyses were obtained from Bhattacharya et al. (2023). BrainMeta eQTL data are available via the SMR web portal (https://yanglab.westlake.edu.cn/smr-portal/). Cell-type-specific cis-eQTL data were obtained from Jang et al. (2026). All custom analysis code and scripts will be made available on GitHub (https://github.com/DaskalakisLab) upon publication.
